# Poly(2‐ethyl‐2‐oxazoline) (POx) as Poly(ethylene glycol) (PEG)‐Lipid Substitute for Lipid Nanoparticle Formulations

**DOI:** 10.1002/smll.202411354

**Published:** 2025-03-19

**Authors:** Caroline T. Holick, Tobias Klein, Charlotte Mehnert, Franziska Adermann, Ilya Anufriev, Michael Streiber, Lukas Harder, Anja Traeger, Stephanie Hoeppener, Christian Franke, Ivo Nischang, Stephanie Schubert, Ulrich S. Schubert

**Affiliations:** ^1^ Laboratory of Organic and Macromolecular Chemistry (IOMC) Friedrich Schiller University Jena, Germany, Humboldtstraße 10 07743 Jena Germany; ^2^ Jena Center for Soft Matter (JCSM) Friedrich Schiller University Jena, Germany, Philosophenweg 7 07743 Jena Germany; ^3^ Helmholtz Institute for Polymers in Energy Applications Jena (HIPOLE Jena) Lessingstraße 12–14 07743 Jena Germany; ^4^ Institute of Applied Optics and Biophysics (IAOB) Friedrich Schiller University Jena, Germany, Helmholtzweg 4 07743 Jena Germany; ^5^ Helmholtz‐Zentrum Berlin für Materialien und Energie GmbH (HZB) Hahn‐Meitner‐Platz 1 14109 Berlin Germany

**Keywords:** lipid nanoparticles, PEG alternative, poly(2‐ethyl‐2‐oxazoline), POx‐lipids

## Abstract

Polyoxazolines have long been considered as promising alternatives to poly(ethylene glycol) (PEG) due to their comparable properties, in particular regarding their stealth effect toward the immune system. Lipid nanoparticles (LNPs), as utilized, e.g., in the COVID‐19 vaccines, contain PEG‐lipids. However, alternatives are required because of the “PEG dilemma” recognized by an increase in anti‐PEG antibodies in the human population. In this study, poly(2‐ethyl‐2‐oxazoline) (PEtOx)‐based lipids with different degrees of polymerization are synthesized and subsequently used to formulate mRNA‐loaded LNPs. The effect of polymer chain length on the size, immunoreaction, and transfection efficiency is investigated in detail. In addition, in‐depth transfection studies are performed using super‐resolution microscopy (SRM) to investigate the uptake mechanism of PEtOx‐based LNPs in comparison to PEG‐LNPs. These combined approaches are utilized to identify the best performing LNP, being superior to the commercial PEG‐lipid used in the Comirnaty formulation.

## Graphical abstract

Poly(2‐ethyl‐2‐oxazoline) (PEtOx)‐based lipids with varying degrees of polymerization have been synthesized and formulated into lipid nanoparticles (LNPs). These PEtOx‐based LNPs have been investigated in terms of their size, morphology, and in vitro performance. It is demonstrated that they have the potential to replace poly(ethylene glycol) (PEG) formulations, as they possess similar characteristics to PEG‐based LNPs, and even outperform them.

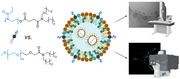

## 1 Introduction

Gene therapy has an enormous potential for the treatment of various diseases. This involves the silencing or replacement of mutant genes or the production of therapeutic proteins.^[^ ^1^ ^]^ However, a major challenge of this therapy is the delivery of nucleic acids. Nucleic acids are known to be rapidly degraded by nucleases and impermeable through the lipid layer of the cellular membrane.^[^ ^2^, ^3^ ^]^ To overcome these challenges, the nucleic acids can be chemically modified or complexes of polymers and genetic material (so‐called polyplexes) can be utilized.^[^ ^4^, ^5^ ^]^ Formulation of lipid nanoparticles (LNPs) can be another effective strategy for transporting nucleic acids.^[^ ^6^ ^]^ Numerous U.S. Food and Drug Administration (FDA) approved LNP‐based drugs have been on the market for almost 30 years, e.g., for the delivery of doxorubicin, but only one transports nucleic acids, in this case siRNA (Onpattro).^[^ ^6^, ^7^ ^]^ In the last few years, two new LNP‐based vaccines, that encapsulate mRNA, i.e., Comirnaty and Spikevax, have been introduced to the market.^[^ ^6^, ^8^ ^]^ The LNPs in these vaccines consist primarily of four lipid components: An ionizable lipid, a neutral phospholipid, cholesterol, and a poly(ethylene glycol) (PEG)‐lipid.^[^ ^6^, ^8^ ^]^ Ionizable lipids not only enhance the biocompatibility in the neutral state, but also ease the endosomal escape upon protonation. In addition, they also interact with the mRNA and can efficiently encapsulate it.^[^ ^9^ ^]^ Phospholipids and cholesterol are primarily incorporated to provide structural stability to the LNPs, while the PEG‐lipid plays a crucial role in controlling particle size, zeta potential, and reducing aggregation. Furthermore, the PEG‐lipid extends the circulation time of the nanoparticles due to its stealth properties.^[^ ^6^ ^]^ However, the abundant usage of PEG in many everyday products, such as cosmetics, already led to an increased formation of anti‐PEG antibodies in the population.^[^ ^10^ ^]^ Blood samples from 2019 revealed that 83% of the donors were positive for anti‐PEG antibodies.^[^ ^11^ ^]^ Moreover, it was observed that patients, who received two doses of the Comirnaty vaccine, have a mean fold increase from 1.8 to 2.6 in the number of PEG‐specific IgG and IgM in the body.^[^ ^12^ ^]^ This leads to an accelerated blood clearance phenomenon, which, among others, reduces the performance of the particles by reduced cellular uptake.^[^ ^13^, ^14^ ^]^ Furthermore, it is known that PEG can cause hypersensitive reactions, which manifest themselves as pseudo allergies. In addition, PEGylation also decreases the cellular uptake and the endosomal release of particles.^[^ ^15^ ^]^ All the disadvantages of PEG are summarized as the “PEG dilemma”.^[^ ^14^ ^]^

This all leads to a significant interest in replacing PEG in nanomedical transport and targeting applications. Polysarcosine (PSar),^[^ ^16^ ^]^ randomized PEG (rPEG),^[^ ^17^ ^]^ which is a PEG derivate with PEG side chains to avoid recognition by antibodies, and polyoxazoline^[^ ^18^ ^]^ represent well‐known alternatives, which are also proposed to replace the PEG‐lipid in the vaccines. Studies using PSar revealed that replacing PEG in LNPs results in reduced immunogenicity and cell toxicity. However, it should be noted that the structural differences between PEG‐ and PSar‐lipids can also affect the performance.^[^ ^14^ ^]^ In a separate study, PSar‐lipids with varying degrees of polymerization (DPs) were tested and the results demonstrated that the systems with higher DP delivered more mRNA to the liver, while the opposite was observed for the spleen.^[^ ^19^ ^]^

Poly(2‐alkyl‐2‐oxazoline)s (POx), particularly those with short alkyl side chains (e.g., methyl or ethyl), have emerged as promising alternatives to polyethylene glycol (PEG) due to their similar stealth properties. Studies have shown that PEG and POx interact with distinct types of proteins, which can influence their biodistribution depending on the target site, while the amount of adsorbed protein was comparable.^[^ ^20^, ^21^, ^22^ ^]^ However, while the stealth effect of PEG is influenced by chain length – longer chains tend to become entangled due to van der Waals forces and interchain hydrogen bonding, reducing their effectiveness – POx exhibits a different behavior.^[^ ^23^ ^]^ In particular, smaller POx polymers (e.g., poly(2‐methyl‐2‐oxazoline) (PMeOx) with a molar mass of 5000 g mol^−1^) are cleared from the body more quickly than their larger counterparts (PMeOx 15 000–30 000 g mol^−1^). This results in a more effective stealth effect for larger POx.^[^ ^24^ ^]^ It has to be noted that the stealth effect for particles can be modified by the density of the stealth polymer grafted on it and compensates the molar mass effect.^[^ ^25^ ^]^ Besides that, POx can also be modified to align with PEG characteristics in terms of their hydrodynamic properties, biocompatibility, hemocompatibility, and hydrophilicity.^[^ ^18^, ^26^, ^27^, ^28^ ^]^ Due to extensive research, a POx‐based prodrug for Parkinson's disease treatment is undergoing clinical trials (NCT02579473).^[^ ^29^, ^30^ ^]^ This could mark the first commercial POx‐based medical product.^[^ ^31^ ^]^

Various strategies have already been developed to replace PEG by POx in LNPs when using functional initiators or terminating agents to form lipid functionalized *α*‐ or *ω*‐end groups, respectively.^[^ ^28^, ^32^ ^]^ Poly(2‐methyl‐2‐oxazoline) (PMeOx) and poly(2‐ethyl‐2‐oxazoline) (PEtOx)‐based lipids with monoacyl lipid end groups were synthesized and compared with the corresponding PEG analogues and DMG‐PEG in LNP formulation. The results demonstrated that monoacyl lipids achieve comparable outcomes with regard to LNP assembly and intracellular delivery.^[^ ^33^ ^]^ In a different study, the synthesis of poly(2‐oxazoline)s was reported, where the *α*‐end group was further modified with lipid end groups. The POx‐lipids were used in the formulation for COVID vaccines and tested in vivo, which revealed that the POx‐lipids have a high potential since the vaccines induce the production of COVID‐19 antigen specific antibodies. It is expected that further modifications of the azide *ω*‐end group of these polymers could enable liver targeting.^[^ ^34^ ^]^ Serina Therapeutics developed *α*‐ and *ω*‐end group functionalized POx‐lipids, which represent an alternative to the PEG‐lipid used in Spikevax. In vivo tests showed a reduced generation of IgM antibodies and, therefore, reduced blood clearance compared to PEG‐based LNPs.^[^ ^35^ ^]^

Here, we present a synthetic approach for PEtOx‐lipids that exhibit structural similarity to the commercial ALC‐0159 (2‐[(polyethylene glycol)‐2000]‐*N*,*N*‐ditetradecylacetamide), the PEG‐lipid utilized in Comirnaty, with the objective of attaining analogous desirable properties. Particular attention was paid to the characterization of these new polymers, since poorly defined polymer structures, in particular in terms of end‐group modifications, may not only have a significant impact on the LNP formulation but also later in the translation into a pharmaceutical product. Within this study, the impact of the chain length on the characteristics of the PEtOx‐based LNPs was examined through the synthesis of a small library comprising five distinct PEtOx DPs between 18 and 99. The PEtOx‐based LNPs were formulated based on the Comirnaty protocol and thoroughly investigated in terms of their size, using dynamic light scattering (DLS) and asymmetric flow field flow fractionation (AF4), encapsulation efficiency, cytotoxicity, transfection efficiency and immunogenicity, including in‐depth in vitro studies. To gain further insight into the cellular uptake of PEtOx‐ and PEG‐based LNPs, additional super‐resolution microscopy studies were conducted.

## 2 Results and Discussion

### 2.1 PEtOx‐Lipid Synthesis

**Scheme 1:**
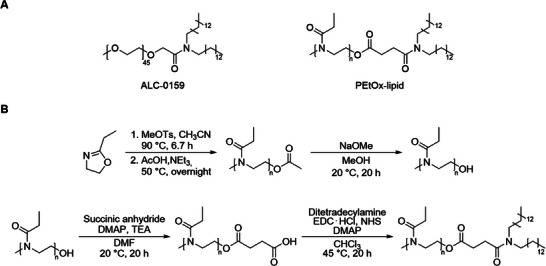
A) Schematic representation of the PEG‐lipid ALC‐0159 used in Comirnaty and the new PEtOx‐lipid. B) Schematic representation of the four‐step synthesis of the PEtOx‐lipid.

**Figure 1:**
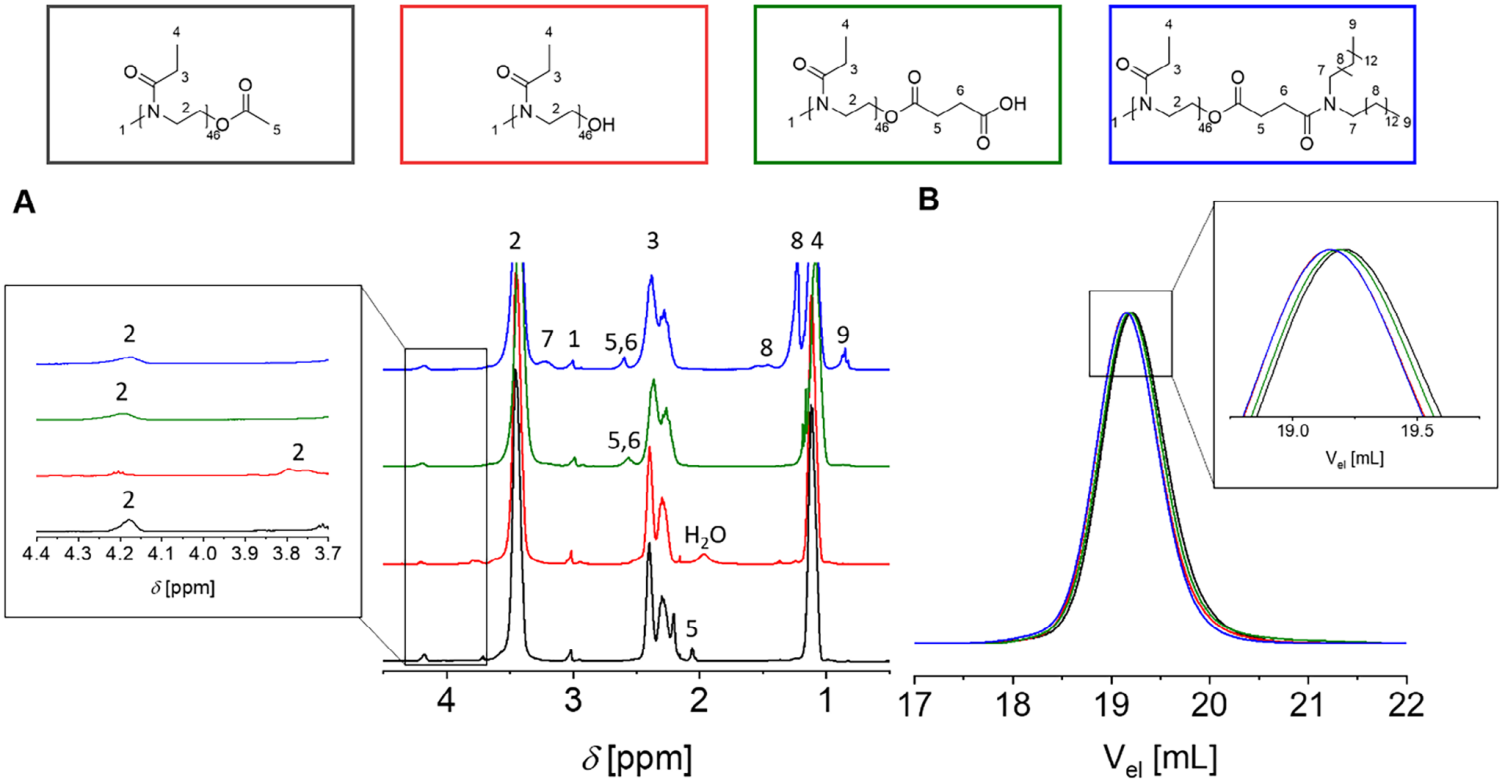
A) ^1^H NMR (CDCl_3_, 500 MHz) overlay of PEtOx_46_‐OAc (black) PEtOx_46_‐OH (red), PEtOx_46_‐COOH (green), PEtOx_46_‐lipid (blue), and zoom in into the last CH_2_ of the polymer backbone. B) SEC overlay of PEtOx_46_‐OAc (black), PEtOx_46_‐OH (red), PEtOx_46_‐COOH (green), PEtOx_46_‐lipid (blue), and zoom in into the tips of the SEC curves. Measured in DMAc + 0.21 wt% LiCl PS calibration.

For the synthesis of an ALC‐0159 analogue, a PEtOx‐lipid four‐step synthesis was established. The initial step was the cationic ring‐opening polymerization (CROP) of ethyloxazoline (EtOx). EtOx can be efficiently polymerized through CROP, enabling a tailored degree of polymerization (DP) and a defined adjustment of *α*‐ and *ω*‐end groups for further functionalization reactions. It is well‐known that the physicochemical character of PEtOx is comparable to PEG, while maintaining the desired stealth effect.^[^ ^27^ ^]^ Methyltosylate was used to initiate the CROP and to introduce a CH_3_ *α*‐end group. The DP was set by the monomer to initiator ratio [M]/[I]. The PEG‐lipid ALC‐0159 contains a PEG with the molar mass of 2000 g mol^−1^, which equals to a DP of 45. As a consequence, the same chain length was chosen for PEtOx. However, previous studies using hydrodynamic techniques have shown that the hydrodynamic volume of PEG is not equivalent to PEtOx with the same DP.^[^ ^18^ ^]^ Therefore, additional polymers with DPs around 45 were synthesized (DP = 38 and 55). To further investigate the effect of chain length, PEtOx with DP = 18 and 99 were also synthesized, resulting in a total set of five polymers with different DPs. The synthesis and characterization of PEtOx‐lipid is exemplarily shown for PEtOx with a DP of 46. The results of polymers with other DP are comparable and can be found in Figures S1–S6 in the Supporting Information. A table with the molar masses determined by proton (^1^H) nuclear magnetic resonance (NMR) spectroscopy, size exclusion chromatography (SEC), and matrix‐assisted laser desorption ionization time‐of‐flight mass spectrometry (MALDI‐TOF MS) as well as the dispersity (*Ð*) values can be found in the Experimental Section (**Table** **6**). The direct termination of the CROP with H_2_O would lead to a nucleophilic attack on the 2‐ and 4‐position of the oxazolinium resulting not only in the formation of PEtOx‐OH but also in a species containing a secondary amine and an ester moiety.^[^ ^36^, ^37^ ^]^ Hence, the CROP of EtOx was terminated first with acetic acid that the living oxazolinium species is formed to an *ω*‐acetate end group resulting in PEtOx‐OAc (**Scheme** **1**).^[^ ^36^ ^]^ The DP of the polymer and the degree of functionalization (DF) were determined by means of ^1^H NMR spectroscopy by comparing the integrals of both end groups (peaks 1 and 2), resulting in a DF of 100% for the acetate functionalization (**Figure** **1**A). The respective molar masses were estimated by SEC. The elugram revealed a polymer with a unimodal and narrow molar mass distribution with a dispersity of ≈*Ð* = 1.05 (Figure 1B).

The synthesis of the hydroxy functionalized PEtOx (PEtOx‐OH) was achieved through the hydrolysis of the acetate group using sodium methanolate. The successful hydrolyzation can be shown via ^1^H NMR spectroscopy by the disappearance of the acetate CH_3_ peak at around 2.1 ppm (Figure 1A). The successful end group modifications can be demonstrated by the shift of the peak of the second CH_2_ group in the last repeating unit of the polymer backbone (Figure 1A, zoom in, peak number 2). Compared to PEtOx‐OAc, the SEC elugram of PEtOx‐OH is slightly shifted to lower elution volumes, caused by the slightly enhanced hydrodynamic volume of the more hydrophilic PEtOx‐OH.

The hydroxy end group was subsequently transformed to a carboxylic acid (PEtOx‐COOH) by using succinic anhydride and dimethylaminopyridine (DMAP) as a catalyst for later coupling of the lipid end group ditetradecylamine. Similar modifications have already been done, where glutaric anhydride was used to incorporate a *ω*‐COOH end group into POx or with succinic anhydride and poly(sarcosine).^[^ ^16^, ^38^, ^39^ ^]^ This ester linker leads to a more degradable structure, separating the polymer from its *ω*‐end group. The successful modification of the end group is evidenced by peaks at 2.48–2.77 ppm in the ^1^H NMR spectrum (Figure 1A, green NMR spectrum, peak 5 and 6), which belong to the two CH_2_ groups of the ester linker. By the shift of the peak of the backbone CH_2_ group from 3.75 to 4.19 ppm, the modification was also proven (Figure 1A, zoom in, peak number 2). The SEC curve reveals no significant difference as PEtOx‐COOH has a similar hydrodynamic volume as PEtOx‐OH. The carboxylic acid was then activated with *N*‐hydroxysuccinimide (NHS) and 1‐ethyl‐3‐(3‐dimethylaminopropyl)carbodiimide (EDC) and allowed to react in situ with ditetradecylamine via amidation. The DF was determined to be 98% via ^1^H NMR spectroscopy. SEC analysis further revealed a narrow distribution with a dispersity of *Ð* = 1.06. For all synthesized polymers, the dispersity did not exceed *Ð* = 1.07, with the exception of PEtOx_99_, which exhibited a higher dispersity of around 1.2. This is a common observation when synthesizing POx with higher molar masses.^[^ ^18^ ^]^

**Figure 2:**
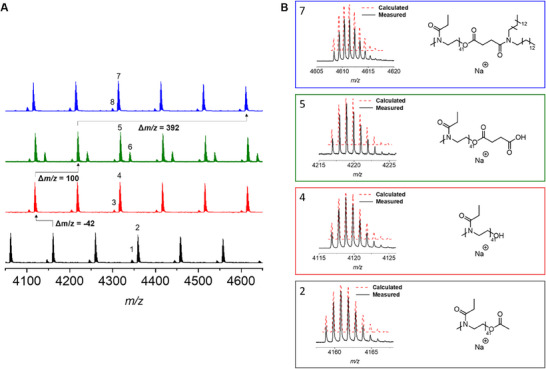
A) Overlay of the MALDI spectra of PEtOx‐OAc (black), PEtOx‐OH (red), PEtOx‐COOH (green), and PEtOx‐lipid (blue). B) Overlay of the calculated isotopic pattern and the measured pattern and the respective main species.

The synthesized polymers were also analyzed by matrix‐assisted laser desorption ionization time‐of‐flight mass spectrometry (MALDI‐TOF MS) to confirm the structures, especially the *α*‐ and *ω*‐end groups. The spectrum of PEtOx‐OAc reveals two distributions belonging to sodium adducts (**Figure** **2**): A small one from the side product with a proton *α*‐end group (Figure 2A, isotopic pattern 1) and the desired acetate *ω*‐end group. The main distribution belongs to the envisaged product (Figure 2A,B, isotopic pattern 2). The spectra for PEtOx‐OH featured distributions which can be assigned to the sodium adduct (Figure 2A,B, isotopic pattern 4). In addition, a PEtOx with proton *α*‐end group, as a result of chain transfer reactions during the CROP, could be found (Figure 2A, isotopic pattern 3). Although it is possible to identify this species, it cannot be quantified by MALDI‐TOF MS.^[^ ^40^ ^]^ For the spectrum of PEtOx‐COOH overlapping with the sodium adduct of the main product (Figure 2A,B, isotopic pattern 5), PEtOx‐OH from the previous reaction could be found. Furthermore, the spectrum featured a species belonging to the sodium salt CH_3_[C_5_H_9_NO]_46_COO^−^ as a sodium adduct (Figure 2A, isotopic pattern 6). This might be a result of the addition of NEt_3_ and the subsequent purification with brine and NaHCO_3_. As expected, the spectrum of PEtOx‐lipid showed again the envisaged product as a sodium adduct (Figure 2A,B, isotopic pattern 7) and the proton‐initiated species (Figure 2A, isotopic pattern 8). However, the proton‐initiated species found in the MALDI cannot be quantified by means of ^1^H NMR. The isotopic patterns of the PEtOx‐lipid and all its precursors perfectly fit to the calculated ones, which also confirms their structure.

### 2.2 LNP Formulation and Characterization

The suitability of the PEtOx‐lipids for LNP formulations was investigated with compositions according to the LNP formulation of Comirnaty and compared with the control formulation containing the PEG‐lipid ALC‐0159. The LNP were composed of 46.3 mol% ALC‐0315, 9.4 mol% 1,2‐dioctadecanoyl‐sn‐glycero‐3‐phosphocholine (DSPC), 42.7 mol% cholesterol and 1.6 mol% of PEG‐ or PEtOx‐lipid as well as mRNA encoding enhanced green fluorescent protein with modified uracil (N1‐methylpseudouridine‐5′‐triphosphate) (me1Ψ UTP EGFP). The lipids were dissolved in ethanol and mixed with citrate buffer at pH 4 containing the mRNA at a nitrogen to phosphate (N/P) ratio of 6 using a tube shaker (type Vortex). The LNPs were characterized in terms of size and polydispersity by DLS measurements (**Table** **1**). In general, it was observed that all LNPs formulated with PEtOx and PEG are similar in sizes with hydrodynamic diameters, *d*_h,DLS_, below 200 nm (**Table** 1). All formulated LNPs in this study exhibit polydispersity index (PDI) values below 0.15, indicating common dispersity values. Zeta potential measurements demonstrated nearly neutral values at pH 7.4 (**Table** 1). In comparison to the ALC‐0159 formulation, PEtOx LNPs with DP = 38, 46, and 55 are slightly more negative.

**Table 1:** Hydrodynamic diameters obtained by batch DLS, *d*_h,DLS_, including polydispersity index (PDI) and AF4, *d*_h,AF4_, as well as diameters of gyration, *d*_g,AF4_. The results are means of *n* = 3 formulations and the standard deviation (SD) refers to the deviation for all formulations. For the SD of *d*_h,DLS_ PDI values were considered.

Sample	*d*_h,DLS_ [nm]	PDI	Zeta potential [mV]	*d*_h,AF4_ [nm]	*d*_g,AF4_ [nm]	$\frac{d_{g,\mathrm{AF}4}\;}{d_{h,\mathrm{AF}4}}$
**ALC‐0159**	138 ± 41	0.091	−0.15 ± 1.58	127 ± 18	98 ± 12	0.773
**PEtOx_18_**	177 ± 64	0.132	−0.76 ± 1.03	155 ± 17	125 ± 9	0.810
**PEtOx_38_**	122 ± 41	0.115	−4.38 ± 0.49	103 ± 5	77 ± 4	0.744
**PEtOx_46_**	115 ± 36	0.098	−4.26 ± 0.71	106 ± 25	81 ± 22	0.760
**PEtOx_55_**	122 ± 40	0.110	−4.92 ± 1.14	107 ± 7	84 ± 10	0.782
**PEtOx_99_**	110 ± 39	0.126	−2.57 ± 0.32	100 ± 8	73 ± 10	0.732

**Figure 3:**
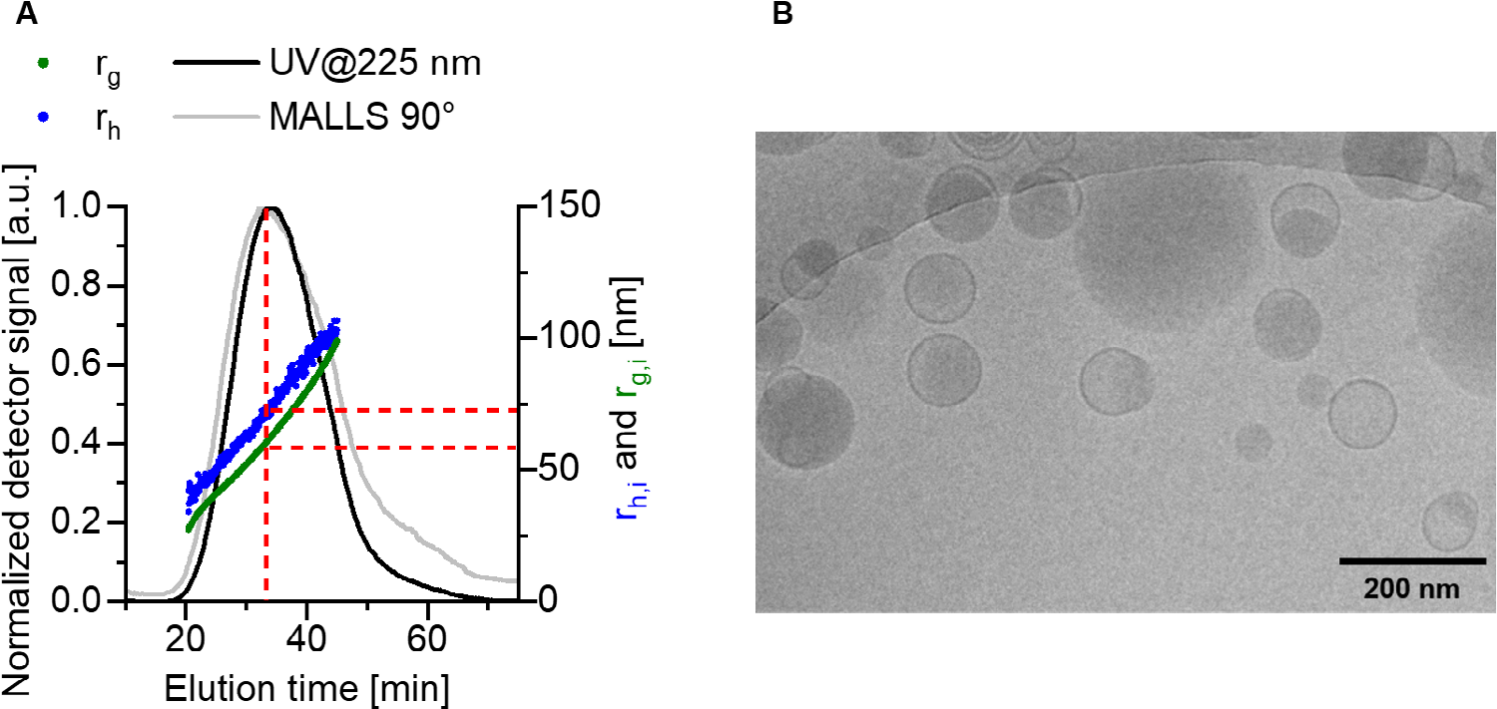
A) Normalized AF4 elution profiles (grey line MALLS@90°, black line UV@225 nm detector) with size traces (blue dots referring to *r*_h,i_, green dots to *r*_g,i_) of PEtOx_18_ LNPs. Red dashed lines indicate the determination of sizes at the maximum of the MALLS@90° elution trace. B) Example zoomed cryo‐TEM image of PEtOx_18_ LNPs (full image can be found in Figure S9 in the Supporting Information).

Next to *z*‐average hydrodynamic diameters that are received by DLS, a more advanced characterization was performed. First, AF4 was conducted with the idea to separate the disperse LNP populations and to perform light scattering on individual elution slices. The AF4 separates the sample according to the diffusion coefficients, whereby faster diffusing species are eluting ahead of slower diffusing species. Therefore, light scattering can be performed on individual elution slices.^[^ ^41^ ^]^ Light scattering on sample slices then provide a more detailed insight on the sample population properties via DLS and multiangle laser light scattering (MALLS). As a result, the radii of gyration *r*_g,i_ (statistical distribution of mass elements around the center of mass of the LNPs), and the hydrodynamic radii, *r*_h,i_, (diffusion‐equivalent hydrodynamic spherical size of the LNPs) can be determined (**Figure** **3**A; Figure S7, Supporting Information). In general, the samples have varying elution profiles and shapes of the population of different batches, while the elution times correlate with the later determined sizes of the particles (Figure S8, Supporting Information). Since the MALLS shows angle‐dependent scattering intensity and the UV@225 nm elution traces are also affected by the Mie scattering effects, there are shifts in the elution profiles against each other (Figures 3A; Figure S7, Supporting Information). Therefore, it is impossible to know the true concentration of LNPs. To have comparability of the here available data within the samples and batches, we always determined *r*_g,i_ and *r*_h,i_ at the maximum of the MALLS@90° elution trace. (Figure 3A, red dashed lines). For comparison, the determined radii are converted to diameters (**Table** 1). Moreover, the ratio of *d*_g,AF4_ and *d*_h,AF4_, i.e., $\frac{d_{g,\mathrm{AF}4}}{d_{h,\mathrm{AF}4}}\;$ indicates the sample anisotropy of the LNPs. The theoretical value for a spherical NP is 0.778.^[^ ^42^ ^]^ For the studied samples, values are in the range from 0.732 to 0.810, which supports the shape of the NPs in the population being spherical.^[^ ^41^ ^]^

The spherical shape was also indicated by cryo‐transmission electron microscopy (cryo‐TEM, Figure 3B; Figure S9, Supporting Information). The images revealed the characteristic bleb structure, which is typically formed when using DSPC in LNP formulation.^[^ ^43^ ^]^ The PEG LNPs and PEtOx_18_ LNPs generally showed more pronounced bleb structures (Figure S10, Supporting Information). It has been also observed that the size of the LNP, as well as its structure, are not uniform. This is in accordance with the range of sizes seen in the DLS and AF4 measurements, and is consistent with those reported for commercial LNPs, for example in the Comirnaty vaccine.^[^ ^44^ ^]^

### 2.3 Immunogenicity Assay and Biological Activity

**Figure 4:**
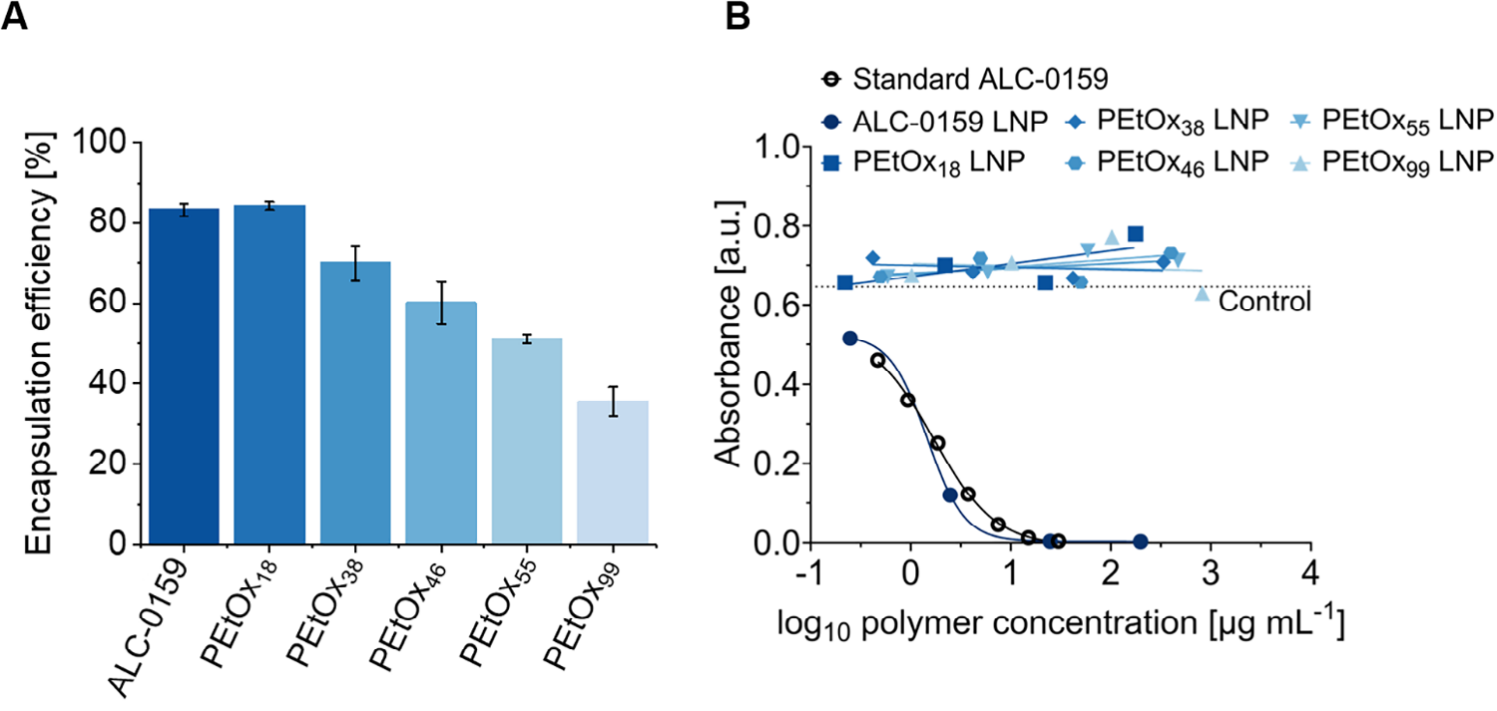
A) Encapsulation efficiency of mRNA in PEG‐ and PEtOx LNPs. Data shown as mean ± SD (*n* = 3 replicates). B) Anti‐PEG antibody specific ELISA as a function of the absorbance against different polymer concentrations. All five PEtOx‐based LNPs as well as the PEG LNP were tested. PBS buffer was used as a negative control. The lipid ALC‐0159 (black circle) was used in known concentrations as the standard in the assay. Data shown as mean (*n* = 2 technical replicates for diluted samples, *n* = 1 for undiluted sample).

The accessible amount of mRNA within the LNPs and their mRNA encapsulation efficiency (EE) was determined using a RiboGreen assay, a fluorescence‐based solution assay.^[^ ^45^ ^]^ The initial mRNA amount used for the formulation was retrievable, indicating no loss of mRNA during the preparation or storage of the LNPs. These results support our proposed formulation procedure, as mRNA is susceptible to degradation by various mechanisms but here protected via the LNP formulations.^[^ ^46^ ^]^ With increasing PEtOx chain length, the EE decreased, resulting in PEtOx_18_ LNPs encapsulating the highest amount of mRNA with a comparable EE to the PEG LNPs (**Figure** **4**A).

The biocompatibility of the novel PEtOx LNPs was assessed through a cytotoxicity screening, in accordance with the international guidelines (ISO 10993–5). The cytotoxicity was measured using adherent mouse fibroblast cells L929, employing the PrestoBlue assay. All LNPs have been tested until a total molar lipid concentration of 500 µmol L^−1^, and no toxic effects were detected in the indicated range (Figure S11, Supporting Information). Furthermore, no significant differences in the cytotoxicity were detected between the PEG LNP and the PEtOx LNPs, highlighting the potential of PEtOx as a viable alternative for LNP formulations. These findings suggest that PEtOx LNPs exhibit favorable biocompatibility, supporting their suitability for further biological investigations, since a high biocompatibility underlines the safety profile of novel and innovative drugs.^[^ ^6^ ^]^

A major problem with conventional LNPs containing PEG is their potential for side effects and increasing body clearance induced by anti‐PEG antibodies. To ensure that the PEG analogues will not induce a possible reaction with these antibodies, an enzyme‐linked immunosorbent assay (ELISA) was performed. Since it was revealed in studies that anti‐PEG antibodies can specifically bind to the PEG backbone, a PEGylated protein ELISA kit containing an antibody specific to the backbone of PEG was used.^[^ ^47^ ^]^ As expected, PEG containing LNPs revealed a strong binding to the PEG antibody while the PEtOx LNPs did not reveal any interaction (Figure 4B). These results clearly demonstrate that PEtOx does not react with anti‐PEG antibodies, thus ruling out the possibility of an autoimmune response due to prior PEG exposure. This effectively circumvents the PEG dilemma and underlines the potential of PEtOx based formulations for patients with pre‐existing anti‐PEG antibodies. It can also be seen that PEG is similarly immunogenic both formulated as a part of LNP and as a lipid itself, which is used here as a standard. It is important to address that repeated exposure to PEtOx may also lead to the production of anti‐PEtOx antibodies in the future, as is currently observed with PEG. Nonetheless, this is a risk associated with any kind of PEG replacement. Therefore, it is crucial to refrain from the use of POx in everyday products such as cosmetics, in order to avoid continuous exposure.

**Figure 5:**
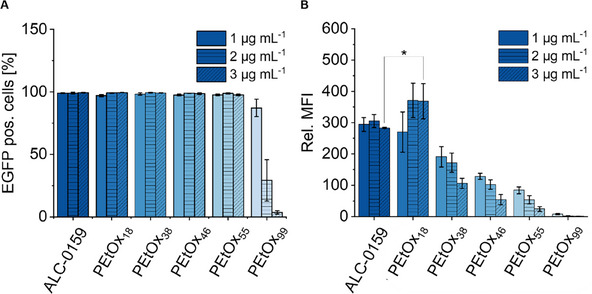
Transfection of HEK293T cells with mRNA loaded LNPs. The transfection efficiency of LNPs in HEK293T cells after 24 h is shown as A) EGFP positive cells and B) mean fluorescence intensity relative to negative control (rel. MFI). Different stealth lipids were used for the respective SLNP. Data shown as mean ± SD (*n* = 3 biological replicates). * *p* ≤ 0.05 derived from a two‐way analysis of variance (ANOVA, further information can be found in the experimental section and Table S1 in the Supporting Information).

The transfection efficacy of the PEtOx‐lipid nanoparticles was investigated in vitro using human embryonic kidney 293 cells (HEK293T) to identify the best performing PEtOx‐lipid for mRNA delivering LNPs. All PEtOx LNPs were tested in comparison to the PEG LNP analogue and the protein expression was measured after 24 h incubation via flow cytometry. For each LNP, three different concentrations of mRNA (1, 2, and 3 µg mL^−1^) were tested. It could be shown that all LNPs were able to transfect cells (**Figure** **5**A) and could, thus, be used for mRNA delivery. Only PEtOx_99_ revealed reduced amount EGFP (enhanced green fluorescent protein) positive cells compared to the other LNPs in particular if higher mRNA concentrations were used. Only 4% EGFP positive cells were detected when 3 µg mL^−1^ mRNA were used (Figure 5A). This effect is even more pronounced if the relative mean fluorescence intensities (rel. MFI) values are considered (Figure 5B). This is in accordance with other studies, where the rel. MFI decreases with higher mRNA concentration when using DSPC.^[^ ^48^ ^]^ Interestingly, a correlation between encapsulation efficiency and protein expression can be observed, although there does not necessarily have to be a causal relationship. However, it would mean that with a higher DP and the same amount of mRNA, more carrier materials would have to be used, which would also increase the amount of lipids/LNPs. Comparing the different PEtOx LNPs with each other, a clear increase in the rel. MFI was detected by decreasing the molar mass of the PEtOx‐lipid. These results are supported by literature reports, where the decrease of the uptake efficiency was correlated to the amount of PEG in LNPs and correspondingly with the increase of stealth effect which is linked to the molar mass of the stealth‐lipid.^[^ ^48^, ^49^ ^]^ The PEG LNP served as positive control, and the best performing PEtOx LNP containing PEtOx_18_ reached significantly higher rel. MFI values at a concentration of 3 µg mL^−1^ compared to the one based on the current commercial standard. Furthermore, the mRNA concentration has little to no impact on the rel. MFI for these two LNPs.

The Comirnaty formulation has demonstrated a proven stability for six months at −80 °C.^[^ ^50^ ^]^ To assess the storage stability of the PEtOx LNPs in comparison to the PEG LNP standard, the particle size, encapsulation efficiency and transfection were evaluated following the at 4 °C for 38 weeks. Notably, the particle sizes of all LNP formulations remained unchanged within the error. The biological properties of the LNPs only revealed little variation within the margin of error (Figure S13, Supporting Information). Detailed information on the size, PDI as well as the encapsulation efficiency can be found (Table S2, Supporting Information). The findings indicate that PEG and PEtOx LNPs remain stable under refrigerated conditions in the absence of added stabilizers.

### 2.4 Uptake and Transfection Kinetic of PEtOx_18_ and PEG LNPs

**Figure 6:**
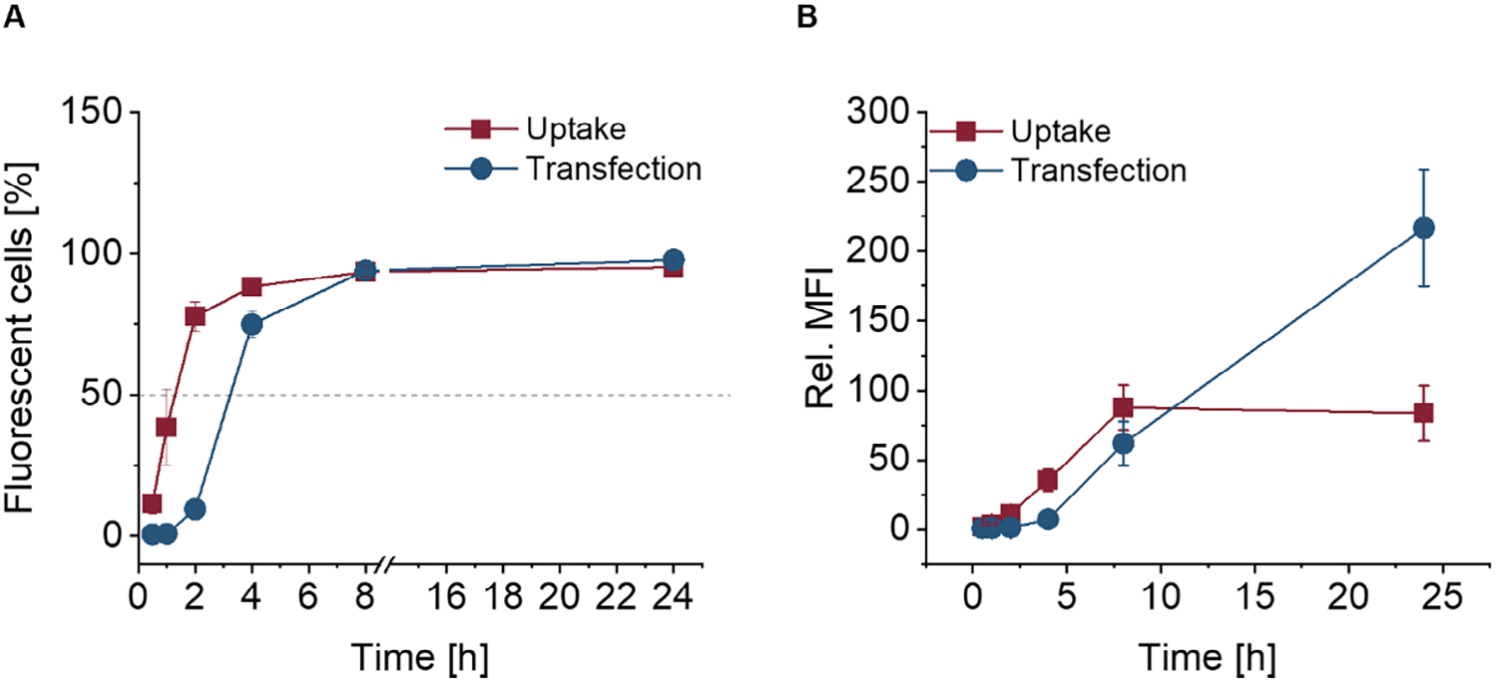
Time dependent uptake (red) and transfection (blue) of PEtOx_18_ LNPs encapsulating Cy5 labeled EGFP mRNA. A) As a function of fluorescent cells against time. B) As a function of the relative mean fluorescence intensities against the time. Data shown as mean ± SD (*n* = 3 biological replicates).

To further investigate the uptake and transfection mechanism of the LNPs, a time dependent kinetic was done with the “best performer” LNPs. Therefore, PEtOx_18_ and PEG LNPs encapsulating Cyanine 5 (Cy5) labeled EGFP mRNA were used with the previous preferred best setup for PEtOx (3.0 µg mL^−1^ mRNA). The LNP exhibits similar particle size and distribution as formulated with me1Ψ‐UTP EGFP mRNA. However, a slightly lower encapsulation efficiency was observed for PEtOx_18_, which might be due to the dye labeled mRNA. The characterization data of the LNPs can be found (Table S3, Supporting Information). HEK293T cells were again transfected with PEtOx_18_‐ as well as PEG LNPs and incubated for up to 24 h. After incubation times of 0.5, 1, 2, 4, 8, and 24 h, the cells were measured via flow cytometry. Already after 0.5 h, an uptake, visualized by the Cy5 positivity of the cells, could be detected (**Figure** **6**A). The uptake increased very fast in the first 2 h, reaching 50% of Cy5 positive cells at 1.2 h, followed by ≈90% of Cy5 positive cells after 4 h and reaching a plateau after 8 h (Figure 6A). This saturation might be explained by a balance between exo‐ and endocytosis or simply by the absence of remaining particles in the media. The transfection and successful production of EGFP is indicated after 2 h of incubation by the detection of 9.5% EGFP positive cells (Figure 6A), and the marker of 50% positive cells is nearly 2 h delayed to the uptake reached at 3.1 h incubation. Focusing on the rel. MFI values, the increase of the transfection signal after 8 h is eightfold higher compared to the 4 h time point (Figure 6B). The transfection reached its maximum at 24 h, while the uptake peaked at 8 h. This behavior underlines the delay between uptake and transfection due to the time dependent translation process of the mRNA. A comparison of PEtOx LNP and PEG LNP, in particular after 2 h, revealed that the tested PEG LNPs were taken up faster (Figures S14 and S15, Supporting Information) resulting in a faster expression of GFP (50% EGFP positive cells after 1.7 h). The observed time dependence and change in total protein level could be explained by the different mRNA used. A change in N1 mψ nucleotide modification to 5 moU and the considerable amount of Cy5‐labeled nucleotides alters the physicochemical properties of the mRNA, which may also influence the interaction within the LNP.

### 2.5 Super‐Resolution Microscopy of PEtOx_18_ and PEG LNPs in Context with Endosomal Cargo

In order to elucidate the intracellular fate of both PEG and PEtOx_18_ LNPs in the context of the endosomal cargos epithelial growth factor (EGF) and transferrin (Tfn), in particular at early time points, we employed multicolor super‐resolution microscopy (SRM). Fluorescently labeled EGF and Tfn have been shown to adequately tag both the endosomal recycling (Tfn) and the degradation (EGF) pathways while not altering the trafficking of LNPs.^[^ ^51^, ^52^ ^]^ In particular, SRM revealed trafficking of EGF and Tfn in small incoming vesicles that arrive at early endosomes via fusion. Subsequently, EGF is sorted from the endosomal membrane to multiple intraluminar vesicles of ≈80 nm diameter. In turn, Tfn is mainly sorted into tubular structures that eventually detach from the endosomal membrane and are recycled to the plasma membrane. Thus, SRM of LNPs in context with these two cargo proteins offers a single‐cell analysis with nanometer resolution and can complement the comprehensive bulk experiments discussed above. For this purpose, co‐uptakes of directly labelled Tfn‐AF488, EGF‐AF555 and LNPs with encapsulated Cy5‐mRNA (further noted as LNPs) were performed, and cells were fixed after 1, 3, 5, 7, 10, 30, 60, and 120 min (see the Experimental Section).

**Figure 7:**
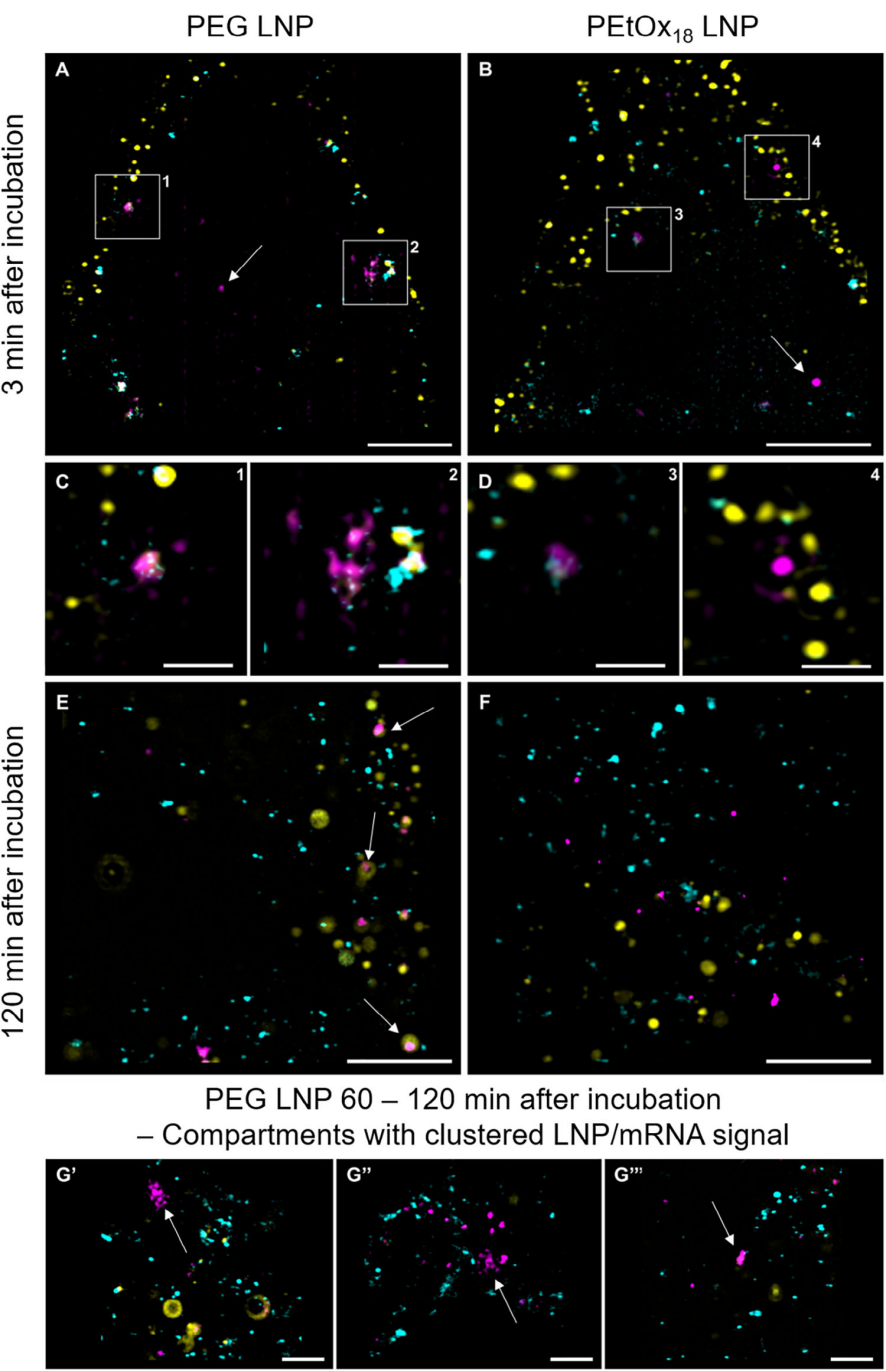
Super‐resolution microscopy of HEK293T cells with mRNA loaded LNPs (magenta) in context with endosomal cargo EGF (yellow) and transferrin (cyan) at different time points after incubation. A–D) In very early time points <5 min after incubation, both the PEG‐ (left column) and PEtOx18‐LNPs (right column) can be detected at the plasma membrane, and already inside cells (indicated by white arrows). Both LNPs can be visualized seemingly co‐localized with endosomal cargo and on their own. E,F) For the PEG LNP large EGF‐positive compartments likely to be lysosomes are detected that incorporate LNP material (white arrows), while for the PEtOx18 LNP these instances are found much less often. G′ – G′′′) For the PEG LNP we find some instances of large, diffuse but spatially concentrated LNP signal, resembling the morphology of arrested endosomes (white arrows). Scale bars: 2 µm (A, B, E, F, G′, G′′, G′′′) and 500 nm (C,D).

Volumetric, triple‐color structured‐illumination microscopy (SIM) imaging of fixed cells at the indicated time points for both the PEG‐ and PEtOx_18_ LNPs were performed first. For very early time points after the incubation, no noticeable difference between both formulations were detected, while a few uptake events in general, i.e., single digit instances per cell at a time could be seen. Of note, for both LNPs, an almost instant uptake of LNPs was detected, as isolated signals deep within cells even after only minutes of incubation were consistently seen (**Figure** **7**A,B; Figures S16 and S17 and Videos S1–S4, Supporting Information). Furthermore, instances of early LNP colocalization with Tfn were visualized, hinting towards co‐uptake, while others are seemingly taken up entirely independent of both cargos (Figure 7C,D; Figures S16 and S17, Supporting Information). These cargo‐independent uptakes could indicate alternate uptake mechanisms such as membrane fusion, which could also explain the instant intracellular localization of LNPs after 3 min.

Interestingly, subtle differences between PEG‐ and PEtOx_18_ LNPs were detected regarding their trafficking and endosomal distribution at later stages, from 30 min onwards. For both LNPs, large, EGF positive vesicles, often devoid of Tfn cargo were detected. Based on their morphology and size, these vesicles might be endo‐lysosomes (Figure 7E, F; Figures S16 and S17, Supporting Information).^[^ ^51^, ^52^ ^]^ For the PEG LNP, mRNA‐Cy5 signal colocalized in these compartments were detected to a lower extend as for the PEtOx_18_ LNP. However, also for the latter case some large, EGF positive vesicles were observed in some of the cells. Most notably, in PEtOx_18_ LNP positive cells singular LNPs, often not colocalized with neither EGF nor Tfn were commonly detected. While for the PEG LNP these instances of isolated LNPs were also found, as well as the previously described seeming lysosomal occurrence, sometimes clustered regions of LNPs and diffuse signals were observed that could indicate cytosolic mRNA material. All in all, a rather diverse landscape of LNP trafficking stages was found in the 30–120 min timepoints for both LNPs, which is consistent with a continued uptake of LNPs and not for a depletion of purely receptor mediated endocytosis.

Endo‐lysosomal compartments with clustered, intense LNP/mRNA signal have been previously reported as arrested endosomes, blocked for acidification, thus not contributing to transfection efficiency.^[^ ^52^ ^]^ Indeed, rare instances of micrometer large vesicle‐like structures with a strong but diffuse PEG LNP/mRNA signal were found (Figure 7G), which is not the case for the PEtOx_18_ LNP. However, although the nanoscale morphology matches with those of arrested endosomes, these instances remain very scarce and are matched with single LNPs in and outside of endosomes.

## 3 Conclusion

We developed a synthetic route toward poly(2‐ethyl‐2‐oxazoline)‐based alternatives to ALC‐0159 via the CROP of EtOx by termination of the polymerization with acetic acid and suitable further post‐polymerization modification. Using this route, PEtOx‐lipids with varying DPs were synthesized, thoroughly analyzed, and consequently formulated into LNPs. All PEtOx‐lipids are able to produce uniform LNPs with size, size distribution, and morphology comparable to PEG‐based LNPs. Furthermore, this study reveals that, in comparison to the PEG LNPs, none of the PEtOx LNPs are immunogenic against backbone‐specific PEG antibodies. PEtOx_18_‐LNPs revealed the most promising results in terms of encapsulation and transfection efficiency and, thus, already exceed PEG LNPs even without any formulation optimization. These results indicate that neither the hydrodynamic volume of the PEtOx‐lipid nor the repeating unit have an impact on the performance of the LNP, but the molar mass, since ALC‐0159 has a molar mass of around 2000 g mol^−1^ (PEtOx_18_‐lipid ≈2200 g mol^−1^). Time‐dependent uptake studies revealed that PEG was taken up more rapidly compared to PEtOx_18_ LNPs. Additionally, early time point uptake mechanisms were demonstrated using super‐resolution microscopy. For both PEG and PEtOx LNPs, uptake events within 1 minute could be observed. Given that LNPs were already localized intracellularly within 3 min, this rapid uptake is, in our opinion, unlikely to be solely explained by receptor‐mediated endocytosis and subsequent escape from endocytic vesicles. While we hypothesize that membrane fusion, which was observed in POx vesicles at early time points,^[^ ^53^ ^]^ may contribute to this phenomenon, further studies would be required to validate this mechanism conclusively, e.g., by inhibiting based on the mechanisms.^[^ ^54^ ^]^ Alternative explanations, such as rapid endocytosis followed by vesicle disruption, can also not be excluded. Lastly, storage stability tests at 4 °C demonstrated that the sizes as well as the transfection efficiency of both PEG and PEtOx LNPs remained unchanged within the experimental error, proving a long shelf‐life stability for both LNPs. As the formulation in this study was based on the Comirnaty vaccine, it is to be expected that with an optimized formulation procedure, the PEtOx LNPs will outperform the PEG standard in the future in terms of, e.g., encapsulation and transfection efficiency, while providing a suitable non‐PEG alternative.

## 4 Experimental Section

### Materials

2‐Ethyl‐2‐oxazoline (EtOx, Sigma Aldrich, ≥99%) was predried over BaO and distilled under inert conditions. Methyl tosylate (MeTos, Sigma Aldrich, 97%) was dried over CaH_2_ and distilled under reduced pressure. Acetic acid (VWR, ACS, Reag. Ph. Eur.), triethylamine (Sigma Aldrich, ≥99%), 0.5 m NaOMe in MeOH (Sigma Aldrich), NHS (Sigma Aldrich), EDC (Sigma Aldrich, ≥97%), ditetradecylamine (Ambeed, 95%), DMAP (abcr, 99%), and succinic anhydride (Sigma Aldrich) were used without further purification. Acetonitrile, methanol, DMF and ethanol were dried in a solvent purification system (SPS, Pure solv EN, InnovativeTechnology).

For the formulation, the following materials were used: EZ Cap Cy5 EGFP mRNA (5‐moUTP) (ApexBio Technology), ALC‐0315 (2‐hexyl‐decanoic acid, 1,1′‐[[(4‐hydroxybutyl)imino]di‐6,1‐hexanediyl] ester (Cayman Chemical), DSPC (1,2‐distearoyl‐sn‐glycero‐3‐phosphocholine (Avanti Polar Lipids), cholesterol (Sigma‐Aldrich), ALC‐0159 (*α*‐[2‐(ditetradecylamino)‐2‐oxoethyl]‐*ω*‐methoxy‐poly(oxy‐1,2‐ethanediyl)) (Cayman Chemical), citrate buffer (Thermo Fisher, diluted with UltraPureTM DNase/RNase‐free distilled water), PBS buffer (Alfa Aesar, diluted with UltraPureTM DNase/RNase‐free distilled water), Quant‐iT RiboGreen RNA reagent (Thermo Fisher Scientific Inc.), triton X‐100 (Sigma‐Aldrich), heparin sodium salt form porcine intestinal mucosa, IU ≥ 100/mg (Alfa Aesar), tris‐ethylenediaminetetraacetic acid (tris‐EDTA, TE) buffer (AppliChem, adjusted to pH 7.5 from TE buffer (1X) pH 8.0).

The subsequent materials were used for the In vitro transcription (IVT) of me1Ψ‐UTP EGFP mRNA and transfection studies: pCMV‐T7‐EGFP (Addgene), fastDigest BshTI (Thermo Fischer Scientific), highYield T7 ARCA mRNA (me 1Ψ‐UTP) Synthesis Kit (Jena Bioscience), DNase I Set (Zymo Research Europe), poly(A) Tailing Enzyme Testkit (Jena Bioscience), RNA Clean & Concentrator‐25 (Zymo Research Europe), Dulbecco's Modified Eagle Medium (DMEM) Low Glucose (Capricorn Scientific), fetal Bovine Serum Advanced (Capricorn Scientifi), penicillin/streptomycin (Pen/Strep), 100× (Capricorn Scientific), HEPES Buffer Solution (1 m) (Capricorn Scientific), Dulbecco's PBS (1×) (Capricorn Scientific), trypsin‐EDTA (0.25%) in HBSS (1×) with Phenol Red (Capricorn Scientific).

### Instrumentation

Proton nuclear magnetic resonance spectra (^1^H NMR) were measured using a Bruker AC 300 MHz spectrometer and on a Bruker Avance NEO 500 500.18 MHz CPP BBFO Prodigy probehead. The measurement was performed at room temperature using CDCl_3_ as a solvent. The residual non‐deuterated solvent signal was used as a reference. The spectra were baseline corrected using the software SpinWorks.

Matrix assisted laser desorption and ionization time‐of‐flight mass spectrometry (MALDI TOF MS) was carried out on a rapifleX MALDI‐TOF/TOF system from Bruker Daltonics equipped with a smartbeam 3D laser (355 nm wavelength). The spectra were measured in the positive reflector mode. *Trans*‐2‐[3‐(4‐*tert*‐butylphenyl)‐2‐methyl‐2‐propenylidene]malononitrile (DCTB) and 2,5‐dihydroxybenzoic acid (DHB) were used as matrix, and sodium trifluoroacetate was added as a doping salt. The recording was performed using manufacture's software flexControl 4.0. Evaluation and processing of the recorded spectra was done using manufacture's software flexAnalysis 4.0 including baseline subtraction and internal external calibration using poly(methyl methacrylate) (PMMA) standard from PSS (2500, 5000 g mol^−1^).

Size exclusion chromatography (SEC) was conducted on an Agilent 1200 series system equipped with a PSS degasser, a G1310A pump, a G1329A auto sampler, a Techlab oven at 40 °C, a G1362A refractive index detector (RID) and a PSS GRAM guard/30/1000 Å column (10 µm particle size). DMAc with 0.21 wt% LiCl was used as an eluent at a flow rate of 1 mL min^−1^. Polystyrene (PS) standards (400–1 000 000 g mol^−1^) were used.

The LNPs were characterized in terms of size and polydispersity by dynamic light scattering (DLS) measurements using a Zetasizer Ultra (Malvern Panalytical) with a laser wavelength of *λ*  =   633 nm. For each measurement of the size and polydispersity index (PDI), UV cuvettes consisting of polystyrene (Brand) were used. The intensity fluctuations were monitored at a backscattering angle of 173° and at a temperature of *T* = 25 °C. Samples were equilibrated for 30 s and five runs of 30 s acquisition time were performed. A priori, LNPs were purified by ultrafiltration. 10 µL of the solutions were diluted to 100 µL with PBS buffer. Next to the determination of the *z*‐average hydrodynamic diameter (*d*_H_) of the LNPs, the size distribution by intensity was also observed. In order to determine the LNPs zeta potential, 10 µL of the suspension were diluted to 1 mL with PBS buffer and measured three times at 25 °C. Three independent batches were measured.

Asymmetrical flow field‐flow fractionation (AF4) was performed with an AF2000 system from Postnova Analytics GmbH (Landsberg, Germany), based on a previous report.^[^ ^41^ ^]^ The AF4 systems consisted of a tip and a focus pump, a cross‐flow pump module (PN1130), an autosampler (PN5300), a channel oven (PN4020), a UV–vis detector (PN 3242), a multiangle laser light scattering (MALLS) detector (PN3621), and a Zetasizer Nano ZS DLS instrument operated at a backscattering angle of 173° (Malvern Instruments) coupled in series. The channel had a trapezoidal geometry with a nominal height of the spacer of 350 µm. A regenerated cellulose membrane with a molar mass cutoff of 10 000 g mol^−1^ was used as the accumulation wall. All measurements were performed in an aqueous sodium chloride solution (9 g L^−1^) as the eluent. The channel oven and autosampler temperature were set to *T* = 5 °C. Sample elution conditions were adapted from a previous study where nanoparticles in a wide range of sizes were studied.^[^ ^41^ ^]^ In brief, 20 µL of the aqueous sodium chloride lipid nanoparticle (LNP) solutions at a LNP concentration of *c* = 1 mg mL^−1^ were injected. The set injection flow rate was 0.2 mL min^−1^, the focus flow rate was 1.3 mL min^−1^, and the cross‐flow rate was 1.0 mL min^−1^ resulting in a detector flow rate of 0.5 mL min^−1^. After the focusing step with a duration of 3 min, elution began within a 0.2 min transition time of flows, where the cross‐flow was held constant for 0.2 min during elution and then decreased in an exponential fashion (exponent 0.2) to 0.1 mL min^−1^ cross‐flow in 40 min. Afterward, the cross‐flow was held constant at 0.1 mL min^−1^ for 5 min, followed by a linear decrease to zero within 30 min. After elution, a constant tip flow at 0.1 mL min^−1^ flow rate for 10 min was programmed. Then the flows were conditioned for the next run.

CryoTEM investigations were performed on a FEI Tecnai G2 20 platform with a LaB6 filament at 120 kV acceleration voltage. Samples were prepared on Quantifoil grids (R2/2), which were treated with Ar plasma prior to use for hydrophilization and cleaning. For cryo‐TEM investigations, 8.5 µL of the solution were vitrified on Quantifoil grids using a Vitrobot Mark IV system, and liquid ethane was used as cryogen. Samples were transferred to a Gatan 626 cryo holder and were maintained at a temperature < −175 °C during the entire process. Images were acquired with a Mega View (OSIS, Olympus Soft Imaging Systems) or an Eagle 4k CCD camera, respectively. Image processing was performed with ImageJ, where the images were optimized manually for contrast and brightness.

Tecan measurements were conducted on a plate reader Infinite M200 Pro plate reader (Tecan Group).

Flow cytometry measurements were conducted on a CytoFLEX LX, Beckman Coulter device.

Both super‐resolution microscopy modes where performed on a Zeiss Elyra 7 with lattice SIM (laser module: 000000‐0239‐500) microscope. This version of the microscope has a homogeneous sample illumination and camera‐based detection (CMOS). It is equipped with four lasers (405, 488, 561, 641 nm) and a mercury vapor lamp. For the displayed SIM images, a 63× oil‐immersion objective with an NA of 1.4 was used, which is optimized for the use of the in‐house “lattice SIM” mode. In addition, the built‐in Optovar was used with a further magnification of 1.6×, resulting in an optical pixel size of 62 nm. For measurements dSTORM a 63× oil‐immersion TIRF‐objective with a NA of 1.45 was employed. A quad‐band dichroic beamsplitter and emission filter (LBF 405/488/561/642) was used for all measurements. Each SIM reconstruction stems from a multi‐color z‐stack (3D), with a physical layer distance of 110 nm and was recorded in lattice SIM mode, with each layer representing a separate lattice SIM reconstruction of 13 individual images. Each SIM phase was recorded with an exposure time of 100 ms. The optical gratings for generating the structured illumination were selected according to the software default settings. ZenBlack's internal software tool was used to reconstruct the SIM recordings. A baseline cut was not used for the reconstruction. The quality setting was set to precise. Further post processing of the images was performed in ImageJ. The images were optimized manually for contrast and brightness. A plugin from was used for 3D displays.^[^ ^55^ ^]^

dSTORM raw image stacks were taken in standard switching buffer conditions in HiLo mode, such that the optical layer of choice was maximally illuminated. After a brief period of EPI‐illumination to reduce out of focus background, 10 000–30 000 camera frames were recorded until no significant photoblinking could be detected. Multicolor acquisitions were taken in the usual 640, 561, 488 nm order to avoid photobleaching and cross‐talk. Reconstructions were performed in the rapidSTORM software and images are displayed with a final pixel size of 10 nm.

### PEtOx‐Lipid Synthesis—General Procedure PEtOx‐OAc Synthesis

In a predried flask, which was degassed by a continuous argon stream, methyl tosylate and 2‐ethyl‐2‐oxazoline were dissolved in anhydrous acetonitrile. The reaction was stirred under reflux for 5–24 h. The reaction was terminated by adding acetic acid and triethylamine after 10 min. The reaction was allowed to stir at 50 °C overnight. Finally, the mixture was diluted with chloroform, extracted twice with chloroform and washed twice with aq. NaHCO_3_ and once with brine. The combined organic phases were dried over NaSO_4_ and filtered. The solvent was removed under reduced pressure, and the residue was dried at 40 °C in vacuo. The amount of substances and the equivalents can be found in **Table** **2**.

**Table 2:** Detailed Amounts for the Synthesis of the PEtOx‐OAc Library.

Sample	MeOTs [mL|mmol]	EtOx [mL|mmol]	CH_3_CN [mL]	Acetic acid [µL|mmol]	TEA [µL|mmol]
**PEtOx_18_‐OAc**	1.14|7.55	15.3|151.1	22.5	650|11.3	2100|15.1
**PEtOx_38_‐OAc**	0.57|3.78	15.3|151.5	22.5	325|5.68	1049|7.57
**PEtOx_46_‐OAc**	0.46|3.02	15.3|151.1	22.5	260|4.55	839|6.05
**PEtOx_55_‐OAc**	0.38|2.52	15.3|151.1	22.5	216|3.78	699|5.04
**PEtOx_99_‐OAc**	0.23|1.51	15.3|151.1	22.5	130|2.27	419|3.02

**PEtOx_18_‐OAc**: Yield: 14.1 g (94%). ^1^H NMR (300 MHz, CDCl_3_, 25 °C): *δ* = 4.14–4.23 (br, 2H, CH_2_–O–C═O), 3.35–3.54 (br, 70H, CH_2_–CH_2_), 2.22–2.48 (br, 3H, CH_3_–N), 2.03–2.09 (br, 36H, CH_2_ side chain), 1.78 (s, 3H, CH_3_ acetyl end group), 1.04–1.19 (br, 54H, CH_3_ side chain) ppm.

**PEtOx_38_‐OAc**: Yield: 13.3 g (89%). ^1^H NMR (300 MHz, CDCl_3_, 25 °C): *δ* = 4.13–4.27 (br, 2H, CH_2_–O–C═O), 3.32–3.65 (br, 150H, CH_2_–CH_2_), 2.92–3.11 (br, 3H, CH_3_–N), 2.18–2.54 (br, 76H, CH_2_ side chain), 2.03–2.14 (br, 3H, CH_3_ acetyl end group), 1.01–1.24 (br, 114H, CH_3_ side chain) ppm.

**PEtOx_46_‐OAc**: Yield: 13.8 g (92%). ^1^H NMR (300 MHz, CDCl_3_, 25 °C): *δ* = 4.04–4.26 (br, 2H, CH_2_–O–C═O), 3.31–3.67 (br, 182H, CH_2_–CH_2_), 3.00–3.04 (br, 3H, CH_3_–N), 2.17–2.53 (br, 92H, CH_2_ side chain), 2.05–2.14 (br, 3H, CH_3_ acetyl end group), 0.97–1.23 (br, 138H, CH_3_ side chain) ppm.

**PEtOx_55_‐OAc**: Yield: 12.0 g (80%). ^1^H NMR (300 MHz, CDCl_3_, 25 °C): *δ* = 4.15–4.31 (br, 2H, CH_2_–O–C═O), 3.29–3.77 (br, 218H, CH_2_–CH_2_), 2.93–3.14 (br, 3H, CH_3_–N), 2.17–2.59 (br, 110H, CH_2_ side chain), 2.01–2.17 (br, 3H, CH_3_ acetyl end group), 1.00–1.43 (br, 165H, CH_3_ side chain) ppm.

**PEtOx_99_‐OAc**: ^1^H NMR (300 MHz, CDCl_3_, 25 °C): *δ* = 4.10–4.23 (br, 2H, CH_2_–O–C═O), 3.10–3.79 (br, 394H, CH_2_‐CH_2_), 2.88–3.07 (br, 3H, CH_3_–N), 2.09–2.54 (br, 198H, CH_2_ side chain), 1.98–2.08 (br, 3H, CH_3_ acetyl end group), 0.94–1.22 (br, 297H, CH_3_ side chain) ppm.

### General Procedure PEtOx‐OH Synthesis

PEtOx‐OAc was dissolved in anhydrous MeOH, and NaOMe (0.5 m in MeOH) was added under vigorous stirring, which was continued at room temperature overnight. MeOH was removed under reduced pressure and the residue was dissolved in chloroform. The mixture was washed twice with NaHCO_3_ and brine. After drying the combined organic phases over NaSO_4_, the solvent was removed under reduced pressure. The residue was then dissolved in dichloromethane and precipitated in cold Et_2_O (−80 °C) and washed. The solid was dried at 40 °C in vacuo. The amount of substances and the equivalents can be found in **Table** **3**.

**Table 3:** Detailed amounts for the synthesis of the PEtOx‐OH library.

Sample	PEtOx_n_‐OAc [g|mmol]	NaOMe [µL|mmol]	MeOH [mL]
**PEtOx_18_‐OH**	12|6.466	1290|0.65	86
**PEtOx_38_‐OH**	13.0|3.39	680|0.34	80
**PEtOx_46_‐OH**	13.5|2.92	583|0.29	90
**PEtOx_55_‐OH**	11.8|2.13	430|0.22	80
**PEtOx_99_‐OH**	12|1.21	245|0.12	16

**PEtOx_18_‐OH**: Yield: 10.0 g (85%).^1^H NMR (300 MHz, CDCl_3_, 25 °C): *δ* = 3.70 – 3.86 (br, 2H, CH_2_‐OH), 3.16 – 3.61 (br, 70H, backbone), 2.94 – 3.09 (br, 3H, CH_3_‐N), 2.20 – 2.61 (br, 36H, CH_2_ side chain), 0.97 – 1.25 (br, 54H, CH_3_ side chain) ppm.

**PEtOx_38_‐OH**: Yield: 10 g (78%). ^1^H NMR (300 MHz, CDCl_3_, 25 °C): *δ* = 3.70 – 3.87 (br, 2H, CH_2_‐OH), 3.36 – 3.63 (br, 2H, CH_2_‐O‐C = O), 3.16 – 3.68 (br, 150H, backbone), 3.02 – 3.12 (br, 3H, CH_3_‐N), 2.22 – 2.57 (br, 76H, CH_2_ side chain), 1.03 – 1.26 (br, 114H, CH_3_ side chain) ppm.

**PEtOx_46_‐OH**: Yield: 10.9 g (81%). ^1^H NMR (300 MHz, CDCl_3_, 25 °C): *δ* = 3.69 – 3.86 (br, 2H, CH_2_‐OH), 3.29 – 3.69 (br, 182H, backbone), 2.89 – 3.14 (br, 3H, CH_3_‐N), 2.13 – 2.68 (br, 76H, CH_2_ side chain), 0.98 – 1.44 (br, 138H, CH_3_ side chain) ppm.

**PEtOx_55_‐OH**: Yield: 8.1 g (69%). ^1^H NMR (300 MHz, CDCl_3_, 25 °C): *δ* = 3.26 – 3.57 (br, 218H, backbone), 3.00 – 3.07 (br, 3H, CH_3_‐N), 2.22 – 2.47 (br, 110H, CH_2_ side chain), 1.00 – 1.21 (br, 165H, CH_3_ side chain) ppm.

**PEtOx_99_‐OH**: Yield: 9.6 g (80%). ^1^H NMR (500 MHz, CDCl_3_, 25 °C): *δ* = 3.71 – 3.83 (br, 2H, CH_2_‐OH), 3.20 – 3.67 (br, 394H, backbone), 2.99 – 3.07 (br, 3H, CH_3_‐N), 2.18 – 2.52 (br, 198H, CH_2_ side chain), 1.02 – 1.20 (br, 297H, CH_3_ side chain) ppm.

### General procedure PEtOx‐COOH synthesis

**PEtOx‐COOH**: PEtOx‐OH and DMAP were dissolved in anhydrous DMF. Triethylamine (0.1 eq.) and succinic anhydride were added to the mixture and stirred overnight at rt. The reaction mixture was then precipitated in ice cold diethyl ether and redissolved in dichloromethane. Subsequently, the mixture was washed with sat. NH_4_Cl and the combined organic phases were dried over MgSO_4_. The volatiles were removed under reduced pressure and afterwards dissolved in dichloromethane and precipitated in ice cold diethyl ether. The residual solid was dried *in vacuo* overnight. The amount of substances and the equivalents can be found in **Table** **4**.

**Table 4:** Detailed amounts for the synthesis of the PEtOx‐COOH library.

Sample	PEtOx_n_‐OH [g|mmol|eq.]	Succinic anhydride [g|mmol|]	DMF [mL]	DMAP [g|mmol|]	TEA [µL|mmol|]
**PEtOx_18_‐COOH**	4.5|2.48	0.74|7.43	34	0.32|2.60	35|0.25
**PEtOx_38_‐COOH**	3.0|0.75	0.23|2.25	10	0.10|0.79	11|0.08
**PEtOx_46_‐COOH**	4.5|0.90	0.27|2.71	12	0.12|0.95	13|0.09
**PEtOx_55_‐COOH**	3.5|0.64	0.19|1.92	9	0.08|0.67	8.9|0.04
**PEtOx_99_‐COOH**	4.0|0.41	0.16|1.63	6	0.05|0.43	4.11|0.04

**PEtOx_18_‐COOH**: 2.2 g (47%), ^1^H NMR (500 MHz, CDCl_3_, 25 °C): *δ*  =  4.19–4.33 (br, 2H, CH_2_–O–C═O), 3.31–3.64 (br, 70H, backbone), 3.00–3.09 (br, 3H, CH_3_–N), 2.51–2.68 (br, 4H, CO–CH_2_–CH_2_–CO), 2.22–2.50 (br, H, 36CH_2_ side chain), 1.02–1.20 (br, 54H, CH_3_ side chain) ppm.

**PEtOx_38_‐COOH**: 2.6 g (84%), ^1^H NMR (500 MHz, CDCl_3_, 25 °C): 4.12–4.26 (br, 2H, CH_2_–O–C═O), 3.20–3.61 (br, 150H, backbone), 2.92–3.02 (br, 3H, CH_3_–N), 2.44–2.62 68 (br, 4H, CO–CH_2_–CH_2_–CO), 2.14–2.45 (br, 76H, CH_2_ side chain), 0.96–1.13 (br, 114H, CH_3_ side chain) ppm.

**PEtOx_46_‐COOH**: 3.5 g (75%), ^1^H NMR (500 MHz, CDCl_3_, 25 °C): *δ*  =  4.18–4.30 (br, 2H, CH_2_–O–C═O), 3.30–3.64 (br, 182H, backbone), 3.00–3.08 (br, 3H, CH_3_–N), 2.50–2.68 (br, 4H, CO–CH_2_–CH_2_–CO), 2.19–2.49 (br, H, 92CH_2_ side chain), 1.02–1.20 (br, 138H, CH_3_ side chain) ppm.

**PEtOx_55_‐COOH**: 3.2 g (89%), ^1^H NMR (500 MHz, CDCl_3_, 25 °C): *δ*  =  4.17–4.31 (br, 4H, CH_2_–O–C═O), 3.26–3.65 (br, 218H, backbone), 2.98–3.07 (br, 3H, CH_3_–N), 2.49–2.66 (br, 4H, CO–CH_2_–CH_2_–CO), 2.17–2.50 (br, 110H, CH_2_ side chain), 0.92–1.22 (br, 165H, CH_3_ side chain) ppm.

**PEtOx_99_‐COOH**: 3.8 g (95%), ^1^H NMR (500 MHz, CDCl_3_, 25 °C): *δ*  =  4.18–4.32 (br, 2H, CH_2_–O–C═O), 3.22–3.68 (br, 394H, backbone), 3.00–3.08 (br, 3H, CH_3_–N), 2.50–2.69 (br, 4H, CO–CH_2_–CH_2_–CO), 2.20–2.49 (br, H, 198CH_2_ side chain), 0.91–1.21 (br, 297H, CH_3_ side chain) ppm.

### General Procedure PEtOx‐Lipid Synthesis

**PEtOx‐lipid**: Succinylated PEtOx was dissolved in anhydrous CHCl_3_ and DMAP, NHS and EDC‐HCl were added and stirred for 3 h at rt. Ditetradecylamine was added and the mixture was stirred overnight at 45 °C. Subsequently, the mixture was precipitated in ice cold diethyl ether, redissolved in CH_2_Cl_2_ and cooled to −20 °C for 3–5 h. The precipitate was filtered (0.25 µm PTFE filter), precipitated in ice cold diethyl ether and dialyzed for 3 d against EtOH:water (1:1, 1000 Da MWCO dialysis membrane), 2 d against water and subsequently freeze dried. The amount of substances and the equivalents can be found in **Table** **5**.

**Table 5:** Detailed amounts for the synthesis of the PEtOx‐lipid library.

Sample	PEtOx_n_‐COOH [g|mmol]	NHS [mg|mmol]	DTDA [mg|mmol]	EDC [mg|mmol]	DMAP [mg|mmol]	CHCl_3_ [mL]
**PEtOx_18_‐lipid**	2.0|1.04	0.3|2.61	1.71|4.18	0.60|3.13	0.01|0.10	32
**PEtOx_38_‐lipid**	2.5|2.44	0.18|1.53	1.00|2.44	0.35|1.83	7.45|0.06	19
**PEtOx_46_‐lipid**	2.5|0.49	0.81|1.97	0.14|1.23	0.28|1.47	6.00|0.05	15
**PEtOx_55_‐lipid**	2.5|0.45	0.13|1.12	0.73|1.79	0.26|1.34	5.47|0.05	14
**PEtOx_99_‐lipid**	2.3|0.23	0.07|0.57	0.37|0.91	0.13|0.68	2.76|0.02	7

**Table 6:** Molar masses *M*_n_, dispersity values, *Đ*, and the degree of functionalization, DF, obtained by ^1^H NMR, SEC, and MALDI TOF MS for all PEtOx species.

Sample	*M*_n, theo._ ^a)^ [g mol^−1^]	*M*_n, NMR_ ^b)^[g mol^−1^]	DF [%]	*M*_n, SEC_[g mol^−1^]	*Ð*_SEC_	*M*_n, MALDI_ [g mol^−1^]	*Ð*_MALDI_
**PEtOx_18_‐OAc**	1860	1970	106	3790	1.09	2240	1.05
**PEtOx_18_‐OH**	1800	1930	100	4140	1.09	2320	1.05
**PEtOx_18_‐COOH**	1890	1842	140^c)^	–	–	2350	1.08
**PEtOx_18_‐Lipid**	2290	1600	106	6240	1.06	2860	1.10
**PEtOx_38_‐OAc**	3840	3030	103	7960	1.05	3900	1.02
**PEtOx_38_‐OH**	3800	3000	92	8340	1.04	4000	1.07
**PEtOx_38_‐COOH**	3900	3800	90	8080	1.04	–	–
**PEtOx_38_‐Lipid**	4290	4470	95	8880	1.05	3800	1.09
**PEtOx_46_‐OAc**	4630	4230	105	9230	1.04	4530	1.05
**PEtOx_46_‐OH**	4590	3700	88	9430	1.05	4390	1.05
**PEtOx_46_‐COOH**	4690	4190	76	9080	1.05	4270	1.08
**PEtOx_46_‐Lipid**	5080	4650	104	9370	1.06	4800	1.04
**PEtOx_55_‐OAc**	5530	2420	82	10 780	1.03	5070	1.05
**PEtOx_55_‐OH**	5480	3620	90	10 700	1.05	4980	1.07
**PEtOx_55_‐COOH**	5580	3420	45	10 320	1.04	5070	1.05
**PEtOx_55_‐Lipid**	5980	5670	86	11 700	1.04	4770	1.06
**PEtOx_99_‐OAc**	9890	9610	72	18 240	1.18	6050	1.42
**PEtOx_99_‐OH**	9850	10 820	109	16 610	1.17	5470	1.37
**PEtOx_99_‐COOH**	9950	9600	70	16 500	1.18	8220	1.18
**PEtOx_99_‐Lipid**	10 340	15 570	58	19 720	1.11	6460	1.21

^a)^ Calculated from the monomer conversion after the CROP by means of ^1^H NMR;

^b)^ Calculated from the integrals of the backbone peaks;

^c)^ Overlapping of peaks in the ^1^H NMR.

**PEtOx_18_‐lipid**: 0.2 g (0.1%), ^1^H NMR (500 MHz, CDCl_3_, 25 °C): *δ*  =  4.16–4.28 (br, 2H, CH_2_–O–C═O), 3.37–3.57 (br, 70H, backbone), 3.18–3.30 (br, 4H, O–N–CH_2_), 2.94–3.08 (br, 3H, CH_3_), 2.58–2.67 (br, 4H, CO–CH_2_–CH_2_–CO), 2.23–2.48 (br, 76H, CH_2_ side chain), 1.44–1.60 (d, 4H, N–CH_2_–**CH_2_**), 1.23–1.32 (br, 44H, N–CH_2_–CH_2_–**CH_2_**), 1.05–1.18 (br, 165H, CH_3_ side chain), 0.88 (tr, 6H, CH_3_ *ω*‐end group) ppm.

**PEtOx_38_‐lipid**: 1.8 g (68%), ^1^H NMR (500 MHz, CDCl_3_, 25 °C): *δ*  =  4.09–4.21 (br, 2H, CH_2_–O–C═O), 3.24–3.57 (br, 150, backbone), 3.11–3.17 (br, 4H, O–N–CH_2_) 2.88–3.01 (br, 3H, CH_3_), 2.51–2.60 (br, 4H, CO–CH_2_–CH_2_–CO), 2.14–2.42 (br, 76H, CH_2_ side chain), 1.37–1.54 (d, 4H, N–CH_2_–**CH_2_**), 1.13–1.29 (br, 44H, N–CH_2_–CH_2_–**CH_2_**), 0.96–1.12 (br, 114H, CH_3_ side chain), 0.73–0.85 (tr, 6H, CH_3_ *ω*‐end group) ppm.

**PEtOx_46_‐lipid**: 2.1 g (78%), ^1^H NMR (500 MHz, CDCl_3_, 25 °C): *δ*  =  4.09–4.21 (br, 2H, CH_2_–O–C═O), 3.26–3.57 (br, 182H, backbone), 3.11–3.23 (br, 4H, O–N–CH_2_), 2.88–3.01 (br, 3H, CH_3_), 2.51–2.60 (br, 4H, CO–CH_2_–CH_2_–CO), 2.14–2.42 (br, 92H, CH_2_ side chain), 1.36–1.54 (d, 4H, N–CH_2_–**CH_2_**),.13–1.28 (br, 44H, N–CH_2_–CH_2_–**CH_2_**), 0.96–1.13 (br, 138H, CH_3_ side chain), 0.81 (tr, 6H, CH_3_ *ω*‐end group) ppm.

**PEtOx_55_‐lipid**: 2.0 g (75%), ^1^H NMR (500 MHz, CDCl_3_, 25 °C): *δ*  =  4.15–4.26 (br, 2H, CH_2_–O–C═O), 3.31–3.60 (br, 218H, backbone), 3.17–3.30 (br, 4H, O–N–CH_2_), 2.93–3.07 (br, 3H, CH_3_), 2.57–2.67 (br, 4H, CO–CH_2_–CH_2_–CO), 2.21–2.48 (br, 110H, CH_2_ side chain), 1.42–1.60 (d, 4H, N–CH_2_–**CH_2_**), 1.19–1.33 (br, 44H, N–CH_2_–CH_2_–**CH_2_**), 1.02–1.19 (br, 165H, CH_3_ side chain), 0.89 (tr, 6H, CH_3_ *ω*‐end group) ppm.

**PEtOx_99_‐lipid**: 2.2 g (81%), ^1^H NMR (500 MHz, CDCl_3_, 25 °C): *δ*  =  4.10–4.21 (br, 2H, CH_2_–O–C═O), 3.26–3.62 (br, 394H, backbone), 3.12–3.23 (br, 4H, O–N–CH_2_), 2.94–3.02 (br, 3H, CH_3_), 2.52–2.61 (br, 4H, CO–CH_2_–CH_2_–CO), 2.13–2.47 (br, 198H, CH_2_ side chain), 1.36–1.55 (d, 4H, N–CH_2_–**CH_2_**), 1.14–1.27 (br, 44H, N–CH_2_–CH_2_–**CH_2_**), 0.97–1.14 (br, 297H, CH_3_ side chain), 0.81 (tr, 6H, CH_3_ *ω*‐end group) ppm.

### Lipid Nanoparticle Formulation

Lipid nanoparticles were formulated by mixing an organic phase containing the lipids and an aqueous phase containing mRNA at a molar N/P ratio of 6 and a 1:3 volume ratio. Stock solutions of the ionizable lipid ALC‐0315, the phospholipid DSPC, cholesterol and the stealth lipids ALC‐0159 as well as the PEtOx‐lipids were prepared in ethanol and later mixed for each formulation for a total molarity of 15 × 10^−3^ m and a molar fraction of 46.3 mol% ALC‐0315, 9.4 mol% DSPC, 42.7 mol% cholesterol, and 1.6 mol% of PEG‐ resp. PEtOx‐lipids. The required amount of me1Ψ‐UTP EGFP mRNA (0.126 mg mL^−1^) or EZ Cap Cy5 EGFP mRNA (5‐moUTP) respectively was prepared in 50 × 10^−3^ m citrate buffer pH 4. In a 1.5 mL Eppendorf tube the mRNA phase was vortexed using a Vortex 2 (IKA) on max. speed. While still mixing, the lipid phase was directly added and the mixture was vortexed for a total of 15 s. To stabilize the initial LNP formulation it was slowly diluted with the same volume of PBS buffer pH 7.4 as the formulation volume.

The LNPs were purified by ultrafiltration using a 5804 R centrifuge (Eppendorf) in order to remove excess ethanol. They were transferred to an Amicon Ultra‐4 Centrifugal Filter (30 000 g mol^−1^ MWCO) and centrifuged in a swing bucket rotor with 3000 rcf at 4 °C. The particles were washed with PBS buffer 20‐fold of the volume of the diluted formulation. They were centrifuged until the retentate volume in the filter was at or below 150 µL. The LNP formulation was collected. In order to lose as little LNPs as possible, the filter membrane of the Amicon was rinsed with PBS buffer to collect the LNP adhering to the filter membrane and was transferred to the LNP suspension. The volume of the LNP suspension was adjusted to a final volume of 375 µL.

### mRNA Entrapment/Encapsulation Efficiency (EE)

The accessible amount of mRNA within the LNPs and their mRNA encapsulation efficiency was determined using the Quant‐iT RiboGreen RNA reagent. The experimental procedure was performed according to the manufacturer's guideline. Briefly, LNP suspensions were incubated with the Ribogreen reagent in the presence and absence of 5 µL mL^−1^ Triton X‐100 and 100 µg mL^−1^ heparin. The test was performed in Tris‐EDTA (TE) buffer (adjusted to pH 7.5 from TE buffer (1×) pH 8.0). For analysis, a plate reader Infinite M200 Pro plate reader was used at an excitation wavelength of 485 nm and an emission wavelength of 535 nm. Fluorescence intensities were measured for total mRNA bound to Ribogreen dye after release from LNP by Triton X‐100 and heparin as well as unencapsulated mRNA bound to Ribogreen dye in the absence of Triton X‐100 and heparin. All results for the me1Ψ‐UTP EGFP mRNA loaded LNPs were obtained from three independent samples, measured in triplicate.

### PEG ELISA

The lipid nanoparticles were examined with regard to their behavior towards an antibody specific to the backbone of PEG using a PEGylated protein ELISA kit (Enzo Life Sciences, Inc., Farmingdale, NY USA) according to the manufacturer's protocol. A plate reader at 450 nm was used for the analysis.

### In Vitro Transcription (IVT) of me1Ψ‐UTP EGFP mRNA (mRNA)

The mRNA used for encapsulation was synthesized in house by IVT. All commercial IVT kits were used as recommended by the manufacturer with adopted volumes. pCMV‐T7‐EGFP plasmid were digested with FastDigest BshTI. After that HighYield T7 ARCA mRNA (me 1Ψ‐UTP) Synthesis Kit was used to synthesize mRNA by 2 h incubation at 37 °C in qPCR. To remove the plasmid the samples were treated with DNase I. To add poly‐A to the mRNA we used Poly(A) Tailing Enzyme Testkit and incubated the mRNA for 1 h at 37 °C in qPCR. The reaction was stopped by immediate purification with RNA Clean and Concentrator‐25. To obtain the required amount of mRNA for all LNP batches, 3 mg mRNA was pooled. The concentration of the mRNA was measured with Tecan NanoQuant Plate.

### Transfection of HEK293T Cells with mRNA Loaded SLNP

The biological assay is adapted from Richter et al.^[^ ^56^ ^]^ and Solomun et al.^[^ ^57^ ^]^ The HEK293T cells were cultured at 37 °C in humidified 5% v/v CO_2_ atmosphere in Dulbecco's Modified Eagle Medium (DMEM) Low Glucose (1 g L^−1^). The media was supplemented with 10% v/v fetal bovine serum (FBS), 100 U mL^−1^ penicillin and 100 µg mL^−1^ streptomycin (culture medium). For the experiments 0.2 × 10^6^ cells mL^−1^ were seeded in 500 µL culture medium containing 10 × 10^−3^ m HEPES in a 24‐well plate and cultivated for 24 h. 1 h before the treatment the medium got changed to 450 µL fresh culture medium containing 10 × 10^−3^ m HEPES. SLNP prepared as described above got diluted in PBS to a final mRNA concentration of 10, 20, and 30 µg mL^−1^. The calculation was based on the results of the ribogreen assay. Cells were treated with 50 µL of respective LNP to receive final mRNA concentrations of 1.0, 2.0, and 3.0 µg mL^−1^. Cells got additionally treated with 3.0 µg mL^−1^ free mRNA in 50 µL PBS as negative control (MM). After 24 h, the supernatant was transferred to a fresh 24‐well plate. The cells were immediately detached with trypsin‐EDTA, resuspended in the corresponding supernatant, and analyzed by flow cytometry (CytoFLEX LX, Beckman Coulter. 20 000 events got measured and viable single cells got analyzed by forward and sideward scatter (FSC/SSC). Fluorescence was measured at *λ*_Ex_ = 488 nm with a 525/40 nm bandpass filter (FITC channel). Positive cells were identified by gating against the MM. A detailed gating strategy is provided in Figure S12 in the Supporting Information.

### Uptake and Transfection Kinetic Studies

For Uptake and transfection kinetic studied cells were seeded as described above. The cells got treated with LNP containing Cy5‐labeled GFP mRNA (3.0 µg mL^−1^ total mRNA per well) and then incubated for 24 h. The cells got measured after 0.5, 1.0., 2.0, 4.0, 8.0, and 24 h as described above by using an additional 638 nm laser with a 660/10 nm bandpass filter for Cy5 detection. Positive cells were identified by gating against the MM. A detailed gating strategy is provided in Figure S15 in the Supporting Information.

### Statistical Analysis

To determine statistically significant differences, groups were analyzed by analysis of variance (ANOVA). Statistical significance is denoted as follows: ns *p* > 0.05, * *p* ≤ 0.05, ** *p* ≤ 0.01, *** *p* ≤ 0.001, and analysis was conducted using GraphPad Prism software (v. 10.0.2).

## Conflict of Interest

The authors declare no conflict of interest.

## Supporting information



Supporting Information

Supplemental Video 1

Supplemental Video 2

Supplemental Video 3

Supplemental Video 4

## Data Availability

The data that support the findings of this study are available in the supplementary material of this article.
