# Supplementary material for: Poly(2‐ethyl‐2‐oxazoline) (POx) as Poly(ethylene glycol) (PEG)‐Lipid Substitute for Lipid Nanoparticle Formulations
Source: Small. 2025 Mar 19;21(16):2411354. doi: 10.1002/smll.202411354 (PMC12019917; doi:10.1002/smll.202411354)
Supplement: Supplementary file 1 — Supporting Information [file SMLL-21-2411354-s005.docx]

# Supporting Information

# Poly(2-ethyl-2-oxazoline) (POx) as Poly(ethylene glycol) (PEG)‑lipid Substitute for Lipid Nanoparticle Formulations

*Caroline T. Holick, Tobias Klein, Charlotte Mehnert, Franziska Adermann, Ilya Anufriev, Michael Streiber, Lukas Harder, Anja Traeger, Stephanie Hoeppener, Christian Franke, Ivo Nischang, Stephanie Schubert, Ulrich S. Schubert**

Caroline T. Holick, Tobias Klein, Charlotte Mehnert, Franziska Adermann, Ilya Anufriev, Michael Streiber, Anja Traeger, Stephanie Hoeppener, Ivo Nischang, Stephanie Schubert, Ulrich S. Schubert

Laboratory of Organic and Macromolecular Chemistry (IOMC),

Friedrich Schiller University, Jena, Germany

Humboldtstraße 10, 07743 Jena, Germany

Email: ulrich.schubert@uni-jena.de

Caroline T. Holick, Tobias Klein, Charlotte Mehnert, Franziska Adermann, Ilya Anufriev, Michael Streiber, Lukas Harder, Anja Traeger, Stephanie Hoeppener, Christian Franke, Ivo Nischang, Stephanie Schubert, Ulrich S. Schubert

Jena Center for Soft Matter (JCSM), Friedrich Schiller University, Jena, Germany

Philosophenweg 7, 07743 Jena, Germany

Ilya Anufriev, Ivo Nischang, Ulrich S. Schubert

Helmholtz Institute for Polymers in Energy Applications Jena (HIPOLE Jena)

Lessingstraße 12-14, 07743 Jena, Germany

Ivo Nischang

Helmholtz-Zentrum Berlin für Materialien und Energie GmbH (HZB)

Hahn-Meitner-Platz 1, 14109 Berlin, Germany

Lukas Harder, Christian Franke

Institute of Applied Optics and Biophysics (IAOB)

Friedrich Schiller University, Jena, Germany

Helmholtzweg 4, 07743 Jena, Germany


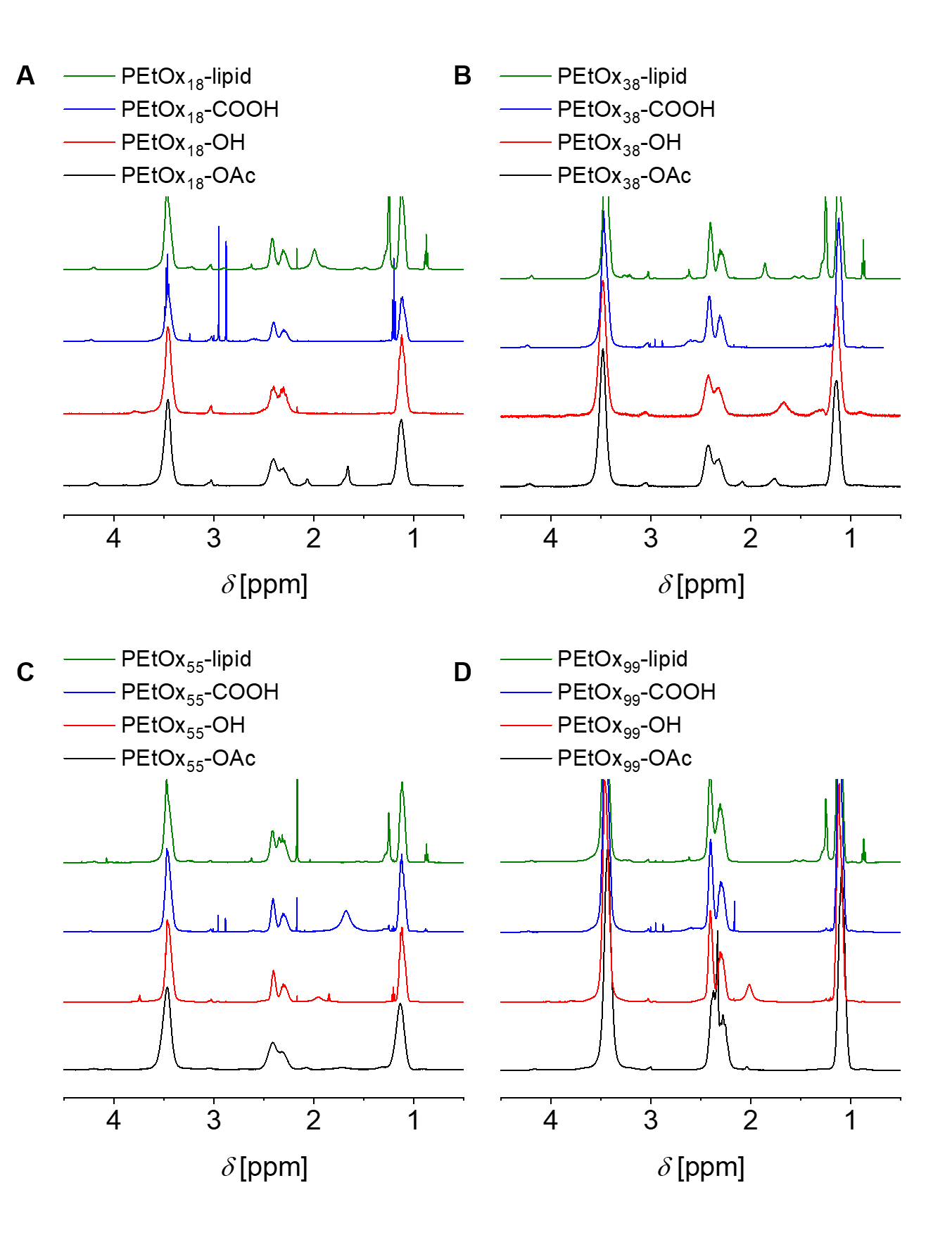


**Figure S 1:** ^1^H NMR spectra overlays for **A**: DP = 18, **B**: DP = 38, **C**: DP = 55 and **D**: DP = 99. PEtOx_n_-OAc (black), -OH (red), -COOH (blue, -lipid (green) (300 and 500 MHz, CDCl_3_).

**Figure S 2:** SEC elugram overlays for **A**: DP = 18, **B**: DP = 38, **C**: DP = 55 and **D**: DP = 99. PEtOx_n_-OAc (black), -OH (red), -COOH (blue), -lipid (green) (CHCl_3_ + iPrOH + NET_3_, PS calibration).


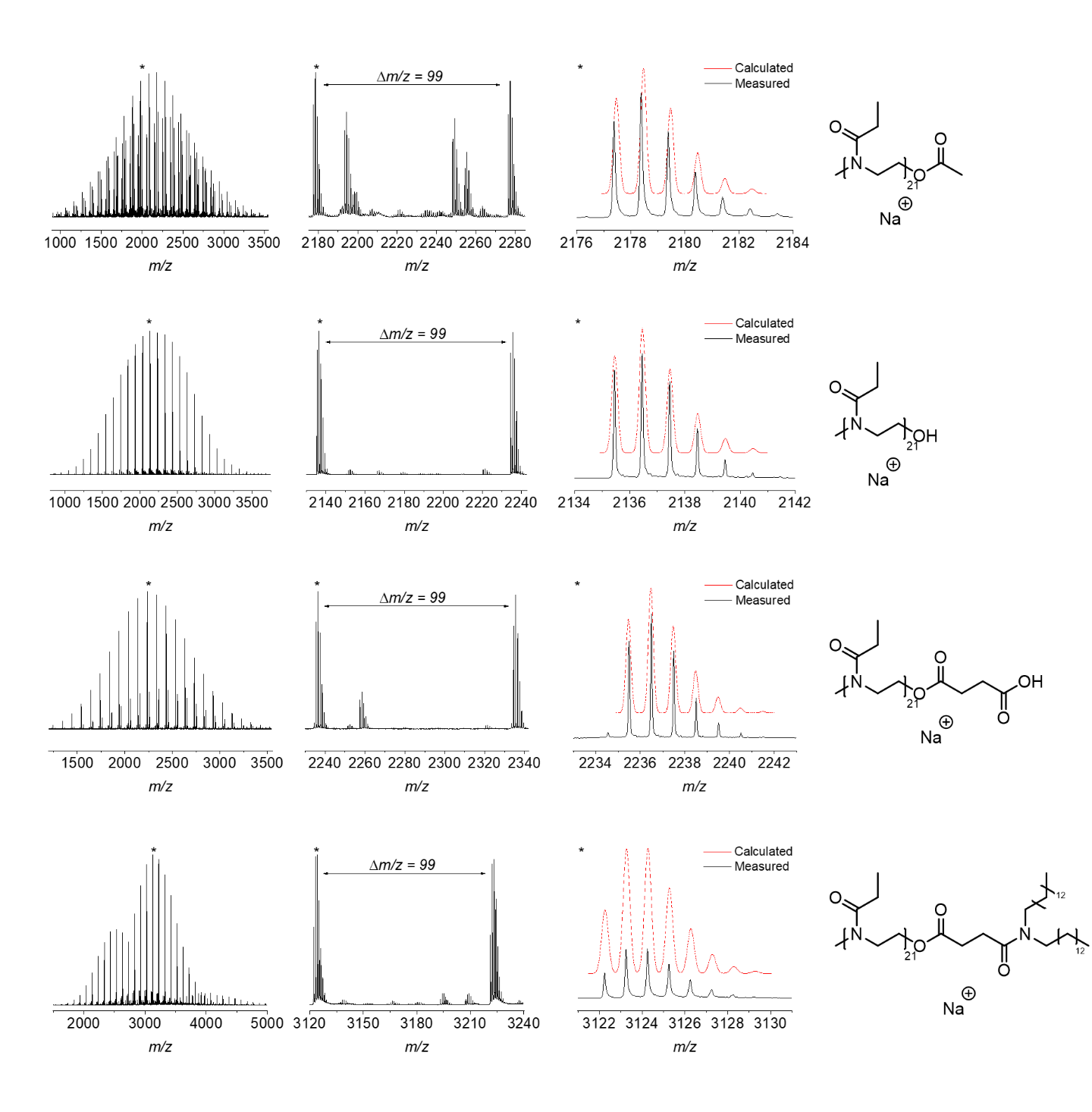


**Figure S 3:** MALDI-TOF MS overlay PEtOx DP = 18. Top to bottom: PEtOx-OAc, PEtOx-OH, PEtOx-COOH and PEtOx-lipid. Left to right: Full spectrum, display of the repeating unit of EtOx Δm/z = 99 and an overlay of the isotopic pattern of the most abundant species (*, black: measured, red: calculated). The identified species were found as sodium adduct.


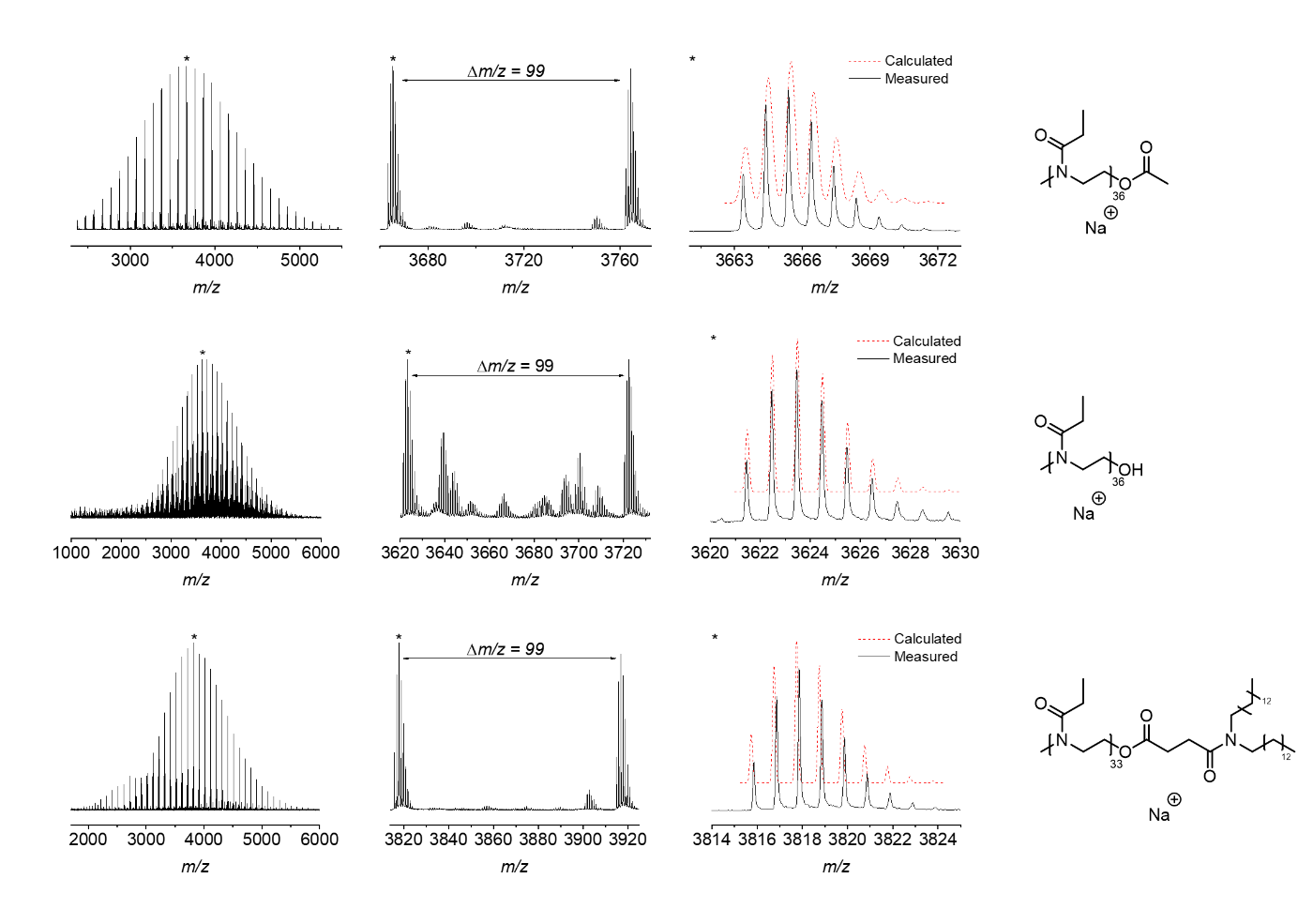


**Figure S 4:** MALDI-TOF MS overlay PEtOx DP = 38. Top to bottom: PEtOx-OAc, PEtOx-OH and PEtOx-lipid. Left to right: Full spectrum, display of the repeating unit of EtOx Δm/z = 99 and an overlay of the isotopic pattern of the most abundant species (*, black: measured, red: calculated). The identified species were found as sodium adduct.


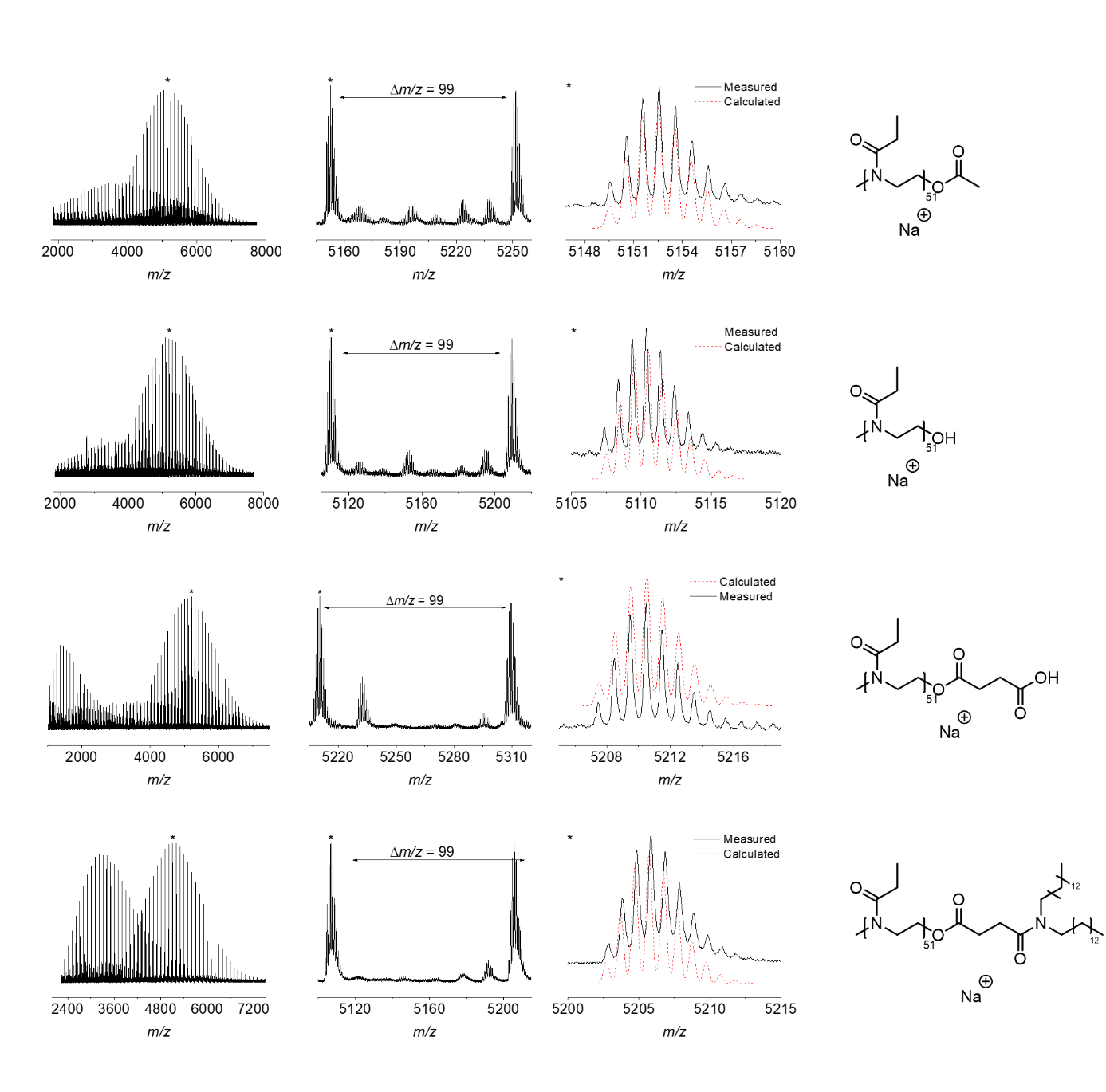


**Figure S 5:** MALDI-TOF MS overlay of PEtOx DP = 55 species. Top to bottom: PEtOx-OAc, PEtOx-OH, PEtOx-COOH and PEtOx-lipid. Left to right: Full spectrum, display of the repeating unit of EtOx Δm/z = 99 and an overlay of the isotopic pattern of the most abundant species (*, black: measured, red: calculated). The identified species were found as sodium adduct.


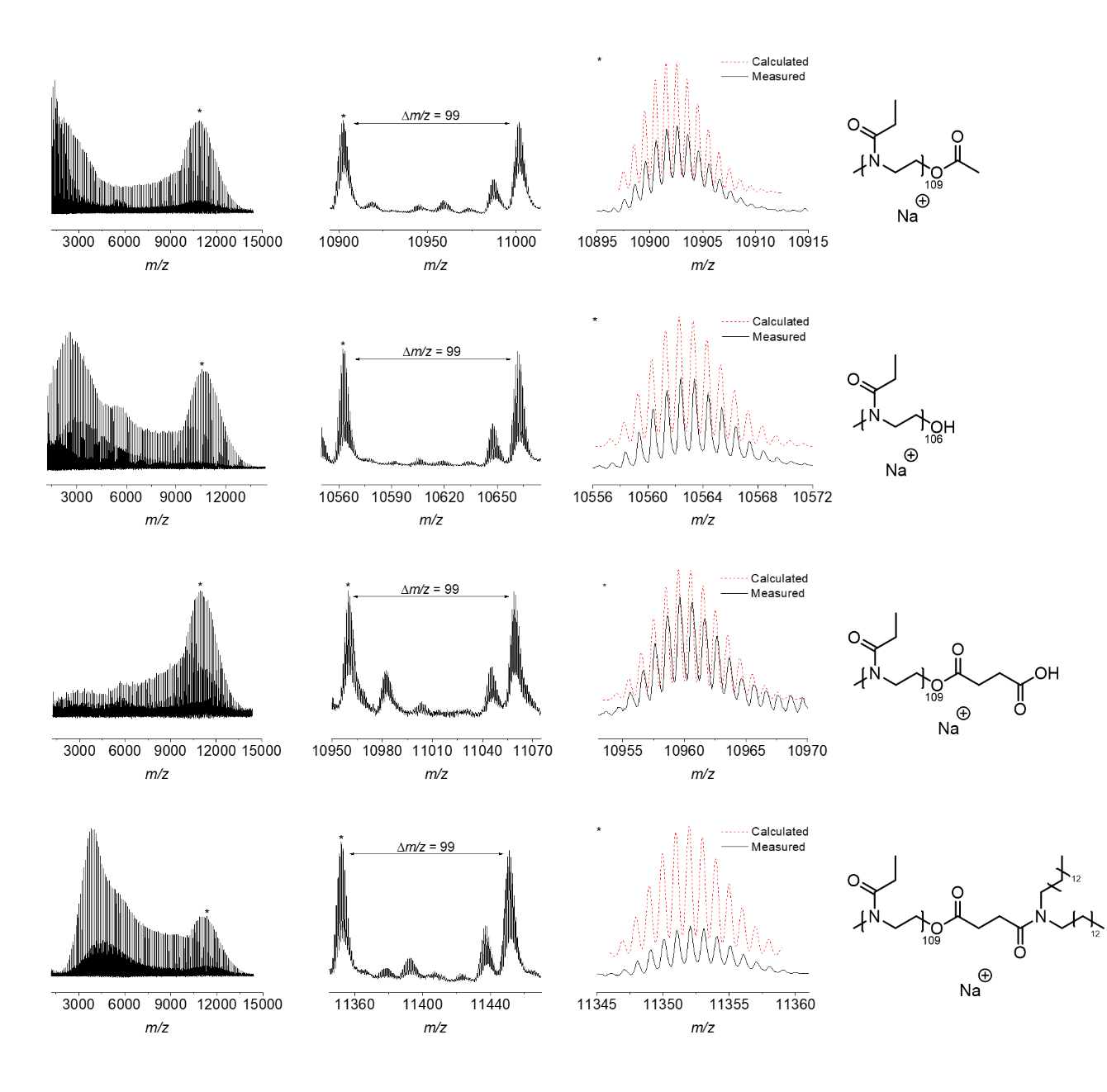


**Figure S 6:** MALDI-TOF MS overlay of PEtOx DP = 99 species. Top to bottom: PEtOx-OAc, PEtOx-OH, PEtOx-COOH and PEtOx-lipid. Left to right: Full spectrum, display of the repeating unit of EtOx Δm/z = 99 and an overlay of the isotopic pattern of the most abundant species (*, black: measured, red: calculated). The identified species were found as sodium adduct.


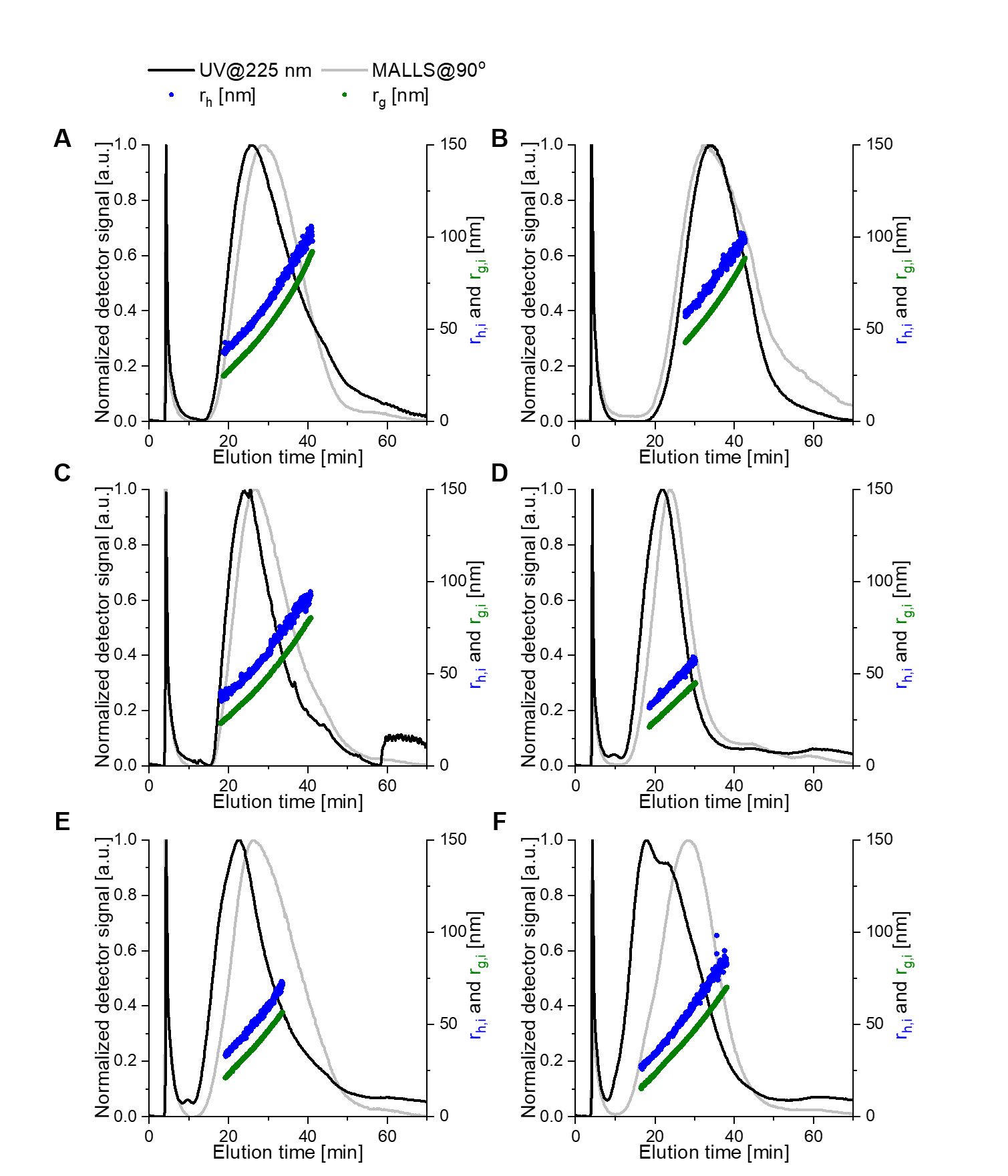


**Figure S 7:** Normalized AF4 elution profiles (black line UV@225 nm, gray line MALLS@90°) with size traces (blue dots referring to r_h,i_, green dots to r_g,i_) of **A**: PEG-LNP, **B**: PEtOx_18_-LNP, **C**: PEtOx_38_-LNP, **D**: PEtOx_46_-LNP, **E**: PEtOx_55_-LNP and **F**: PEtOx_99_-LNP.


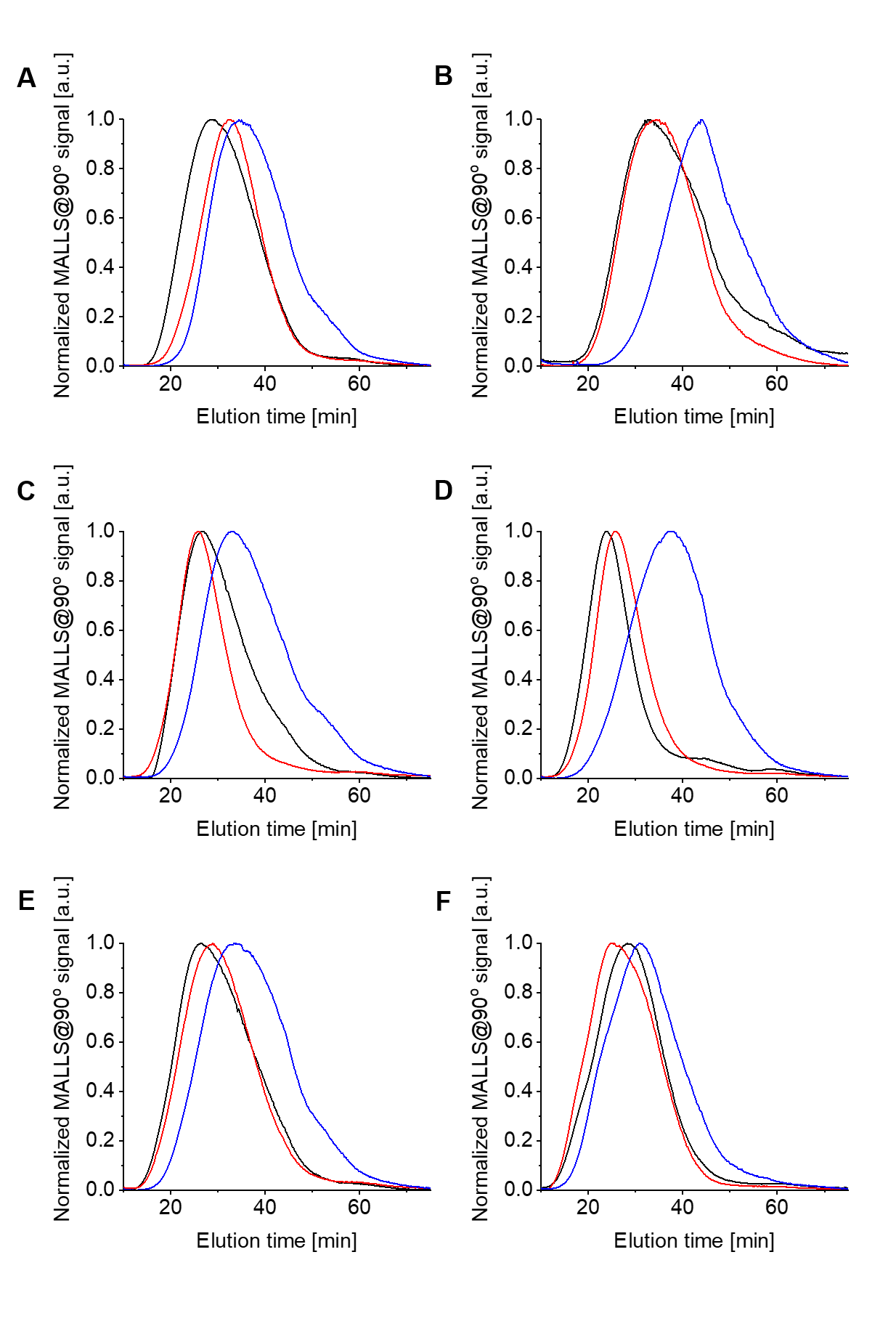


**Figure S 8:** Normalized MALLS@90° detector signals from different batches of **A**: PEG-LNP, **B**: PEtOx_18_-LNP, **C**: PEtOx_38_‑LNP, **D**: PEtOx_46_-LNP, **E**: PEtOx_55_-LNP and **F**: PEtOx_99_-LNP. Black line refers to batch #1, the red line refers to batch #2, and the blue line refers to batch #3.


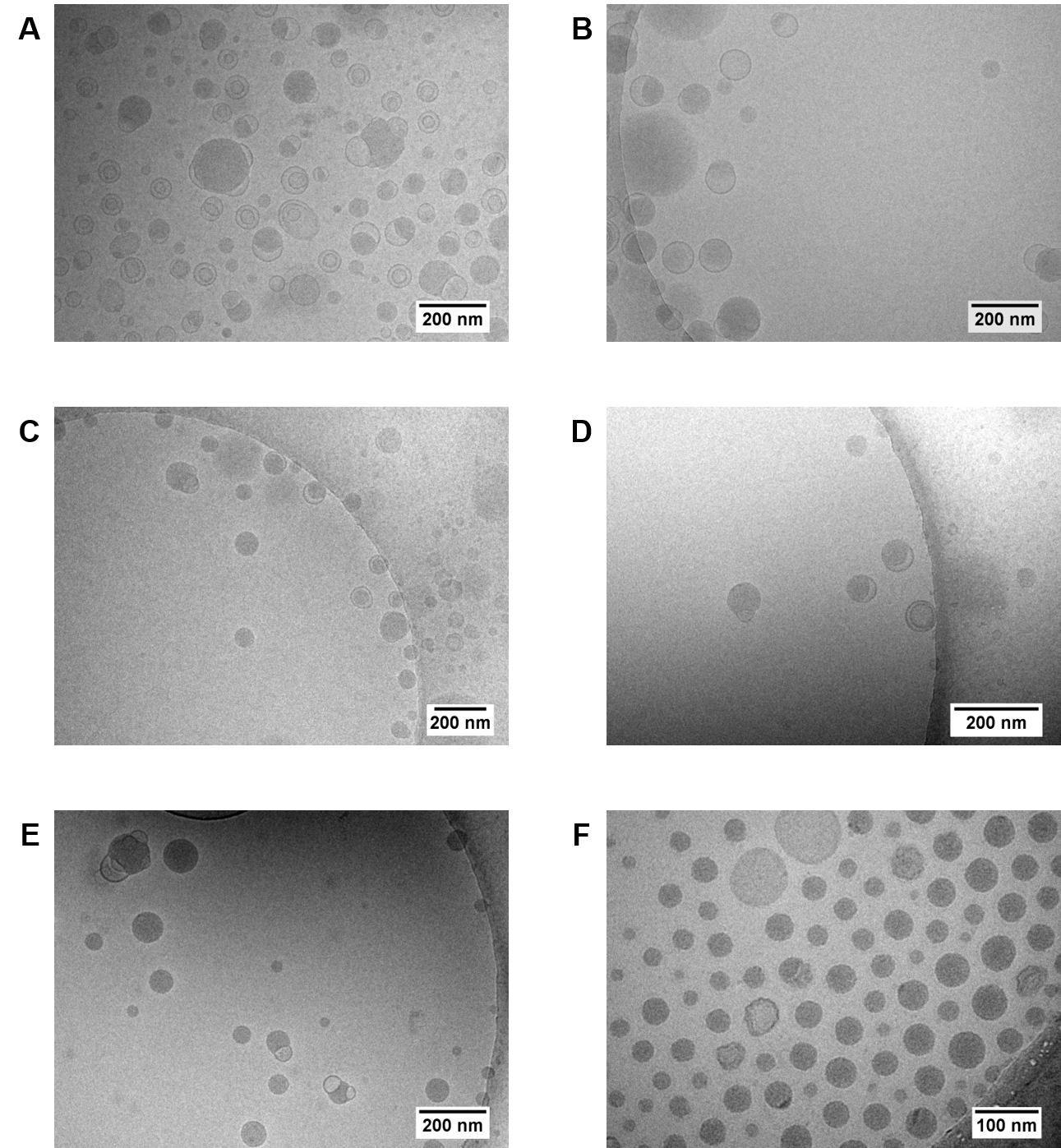


**Figure S 9:** Cryo‑TEM measurements of PEG- and POx-based LNPs. **A**: PEG-LNP. **B**: PEtOx_18_-LNP. **C**: PEtOx_38_-LNP. **D**: PEtOx_46_-LNP. **E**: PEtOx_55_-LNP. **F**: PEtOx_99_-LNP.


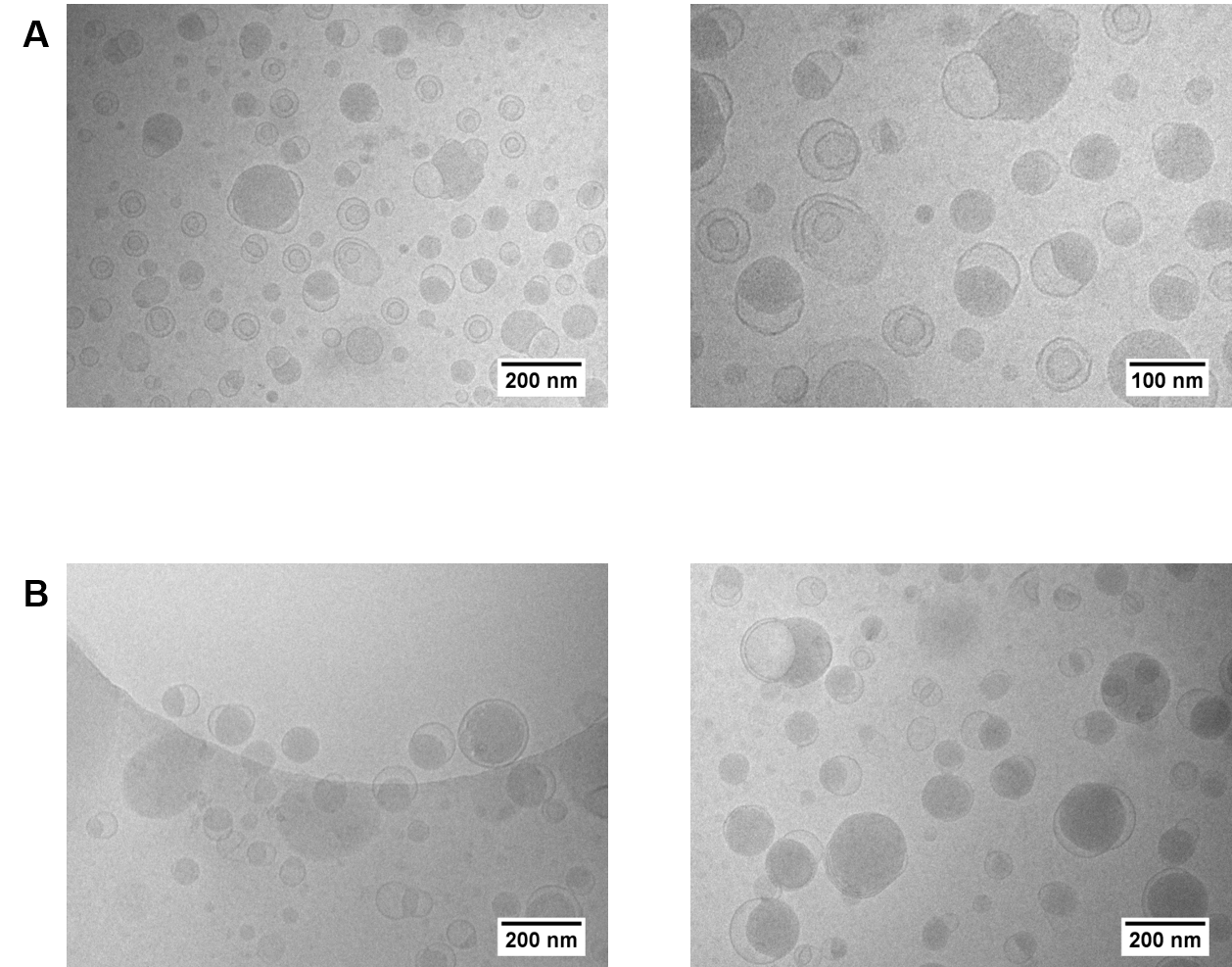


**Figure S 10:** Cryo‑TEM measurements of **A**: PEG-LNP and **B**: PEtOx_18_-LNP.

**Figure S 11:** Cytotoxicity assay (PrestoBlue) of PEG‑ and PEtOx‑based LNPs. Data shown as mean ± SD (n = 3 biological replicates).

**
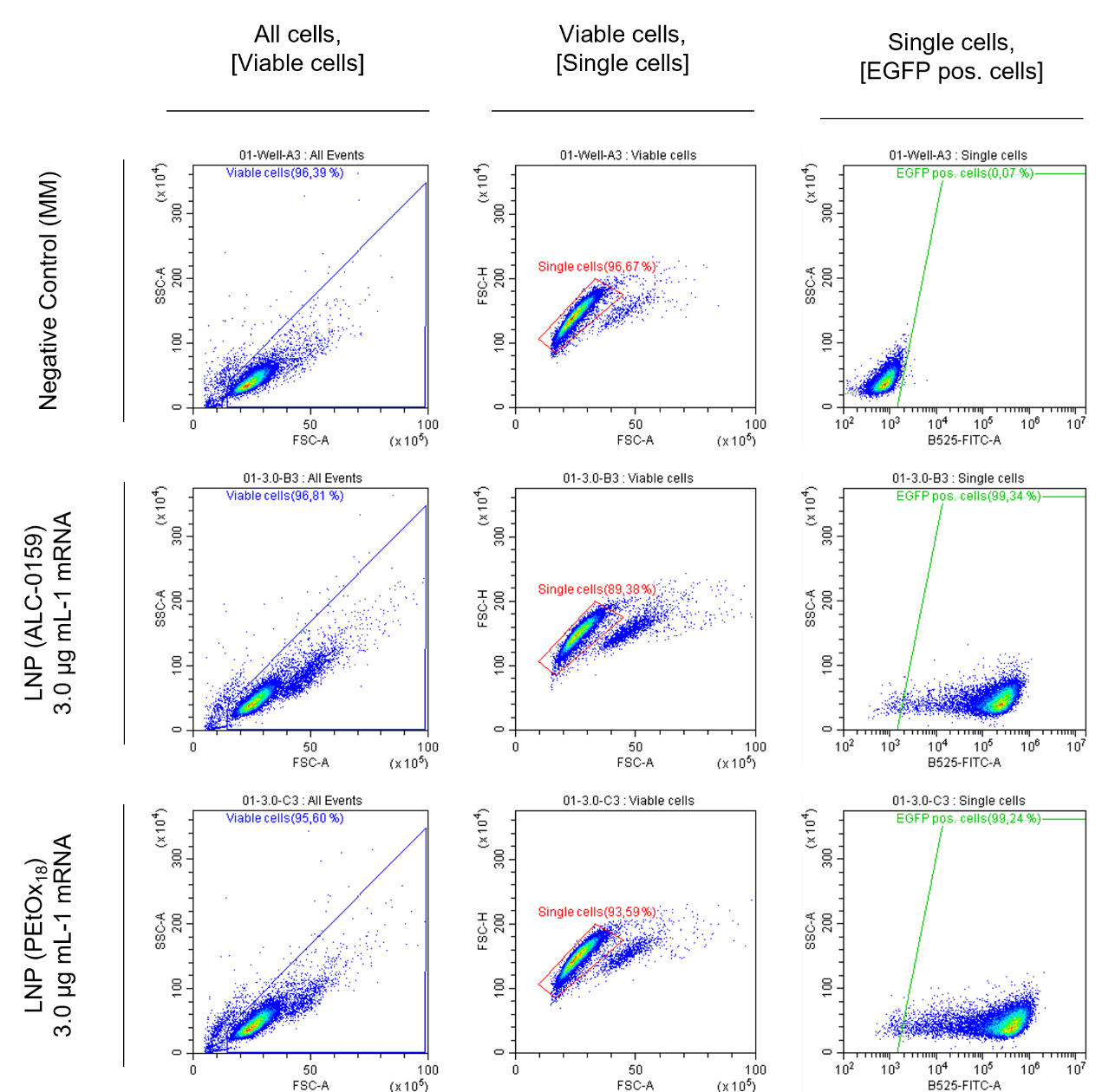
**

**Figure S 12:** Gating strategy: Transfection of HEK293T cells with mRNA (EGFP). Viable single cells were analyzed by forward and sideward scatter (FSC/SSC). Fluorescence was measured at λ_Ex_ = 488 nm with a 525/40 nm bandpass filter (FITC channel). Positive cells were identified by gating against the cells treated with only nucleic acid (MM). Analysis was conducted with CytExpert V 2.5.0.77.

**Table S 1:** Final p values for the transfection.

| Concentration  [µg mL^-1^] |  | Summary | Adjusted P Values |
| --- | --- | --- | --- |
| 1 | ALC-0159 vs. PEtOx_18_-lipid | ns | 0.86 |
|  | ALC-0159 vs. PEtOx_38_-lipid | ** | 0.004 |
|  | ALC-0159 vs. PEtOx_46_-lipid | *** | <.001 |
|  | ALC-0159 vs. PEtOx_55_-lipid | *** | <.001 |
|  | ALC-0159 vs. PEtOx_99_-lipid | *** | <.001 |
| 2 | ALC-0159 vs. PEtOx_18_-lipid | ns | 0.104 |
|  | ALC-0159 vs. PEtOx_38_-lipid | *** | <.001 |
|  | ALC-0159 vs. PEtOx_46_-lipid | *** | <.001 |
|  | ALC-0159 vs. PEtOx_55_-lipid | *** | <.001 |
|  | ALC-0159 vs. PEtOx_99_-lipid | *** | <.001 |
| 3 | ALC-0159 vs. PEtOx_18_-lipid | * | 0.02 |
|  | ALC-0159 vs. PEtOx_38_-lipid | *** | <.001 |
|  | ALC-0159 vs. PEtOx_46_-lipid | *** | <.001 |
|  | ALC-0159 vs. PEtOx_55_-lipid | *** | <.001 |
|  | ALC-0159 vs. PEtOx_99_-lipid | *** | <.001 |

**Table S 2:** Hydrodynamic diameters obtained by batch DLS, d_h,DLS_, polydispersity index (PDI) and the encapsulation efficiency of the LNPs stored at 4 °C for 38 weeks. The results are means of n = 3 formulations and the standard deviation (SD) refers to the deviation for all formulations. For the SD of d_h,DLS_ PDI values were considered.

| Sample | *d*_h,DLS_  [nm] | PDI | EE  [%] |
| --- | --- | --- | --- |
| ALC-0159 | 152±53 | 0.124 | 86±2 |
| PEtOx_18_^a)^ | 187 | 0.219 | 77 |
| PEtOx_38_ | 134±56 | 0.173 | 70±2 |
| PEtOx_46_^a)^ | 123 | 0.189 | 59 |
| PEtOx_55_ | 138±62 | 0.200 | 45±2 |
| PEtOx_99_ | 124^a)^ | 0.166 | 35±2 |

^a)^ *n* = 2, due to insufficient sample availability.


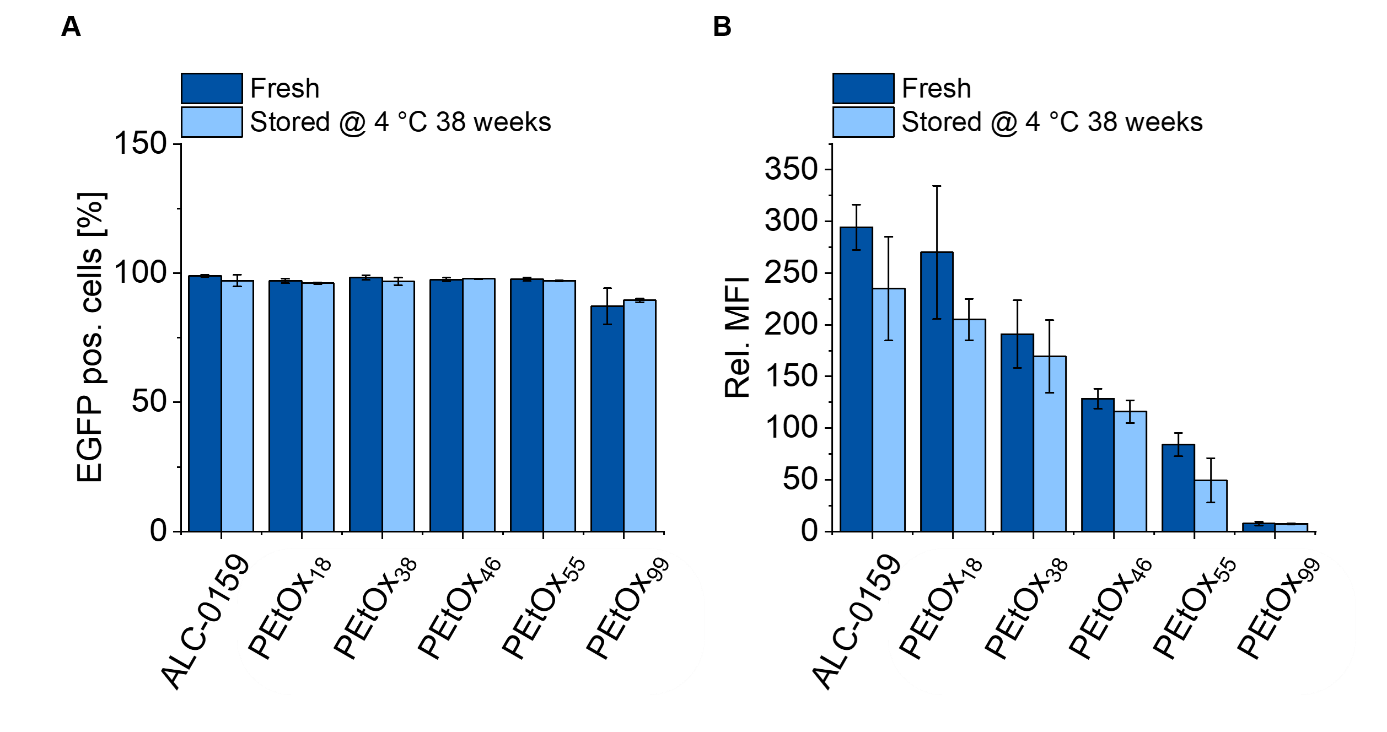


**Figure S 13:** Transfection of HEK293T cells with mRNA loaded LNPs (1.0 µg mL^-1^). **A**: EGFP positive cells after 24 h. **B**: The transfection efficiency of LNPs in HEK293T cells after 24 h is shown as mean fluorescence intensity relative to negative control (rel. MFI). Different stealth lipids were used for the respective SLNP. Dark blue: Freshly formulated particles, same as in **Figure 5**. Light blue: Particles stored at 4 °C for 38 weeks. Data shown as mean ± SD (n = 3 biological replicates).

**Table S 3:** Hydrodynamic diameters obtained by batch DLS, dh_,DLS_, polydispersity index (PDI), zeta potential and the encapsulation efficiency of the LNPs encapsulating Cy5‑GFP labeled mRNA for the uptake and transfection kinetic studies.

| Sample | *d*_h,DLS_  [nm] | PDI | Zeta potential  [mV] | EE  [%] |
| --- | --- | --- | --- | --- |
| ALC-0159 | 132 | 0.082 | -9.75 | 80.0 |
| PEtOx_18_ | 197 | 0.113 | -9.8 | 68.3 |


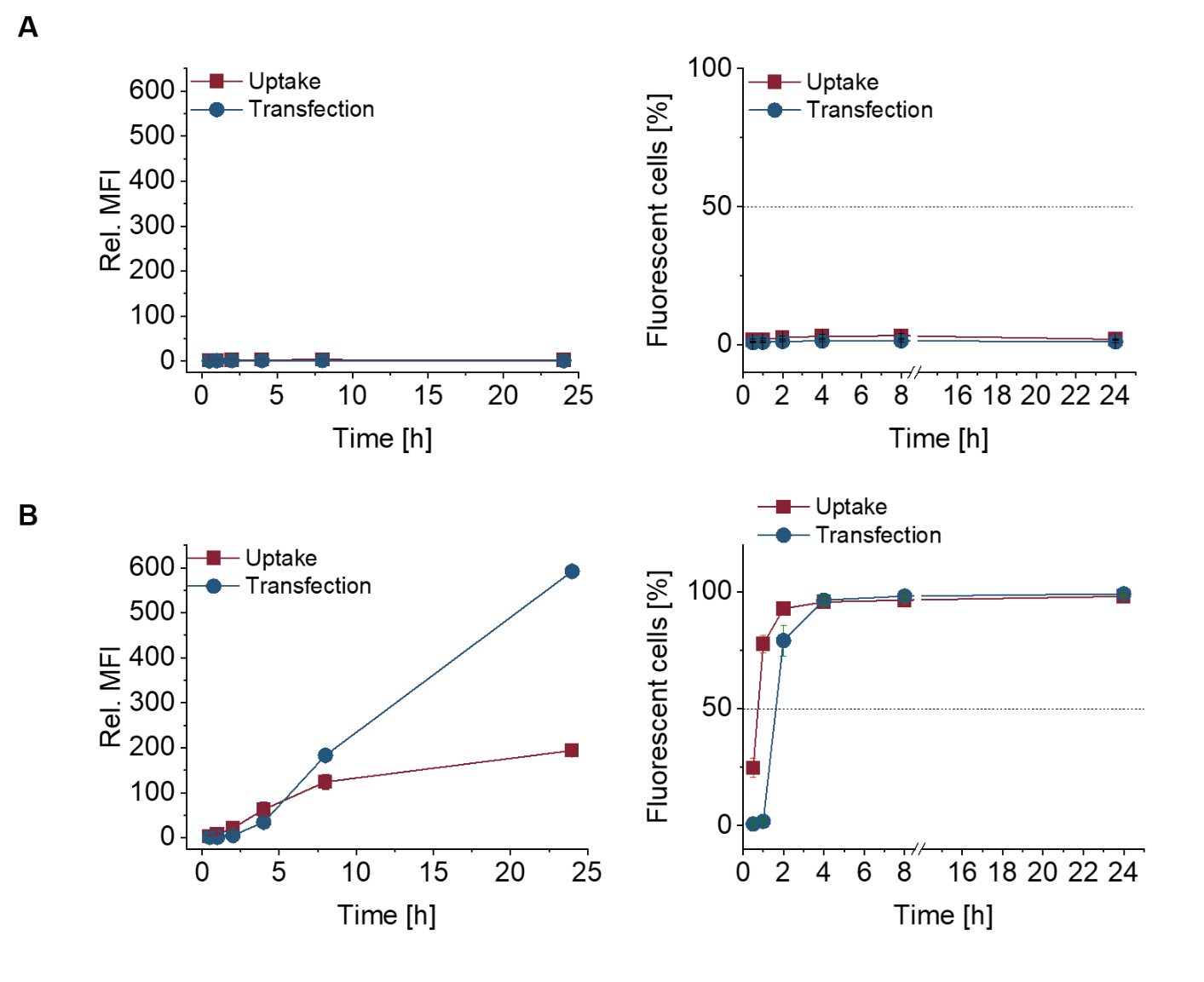


**Figure S 14:** Time dependent uptake (red) and transfection (green) of **A**: the MasterMix and **B**: PEG LNP encapsulating Cy‑5‑GFP labeled mRNA. Left: As a function of the relative mean fluorescence intensities against the time. Right: As a function of fluorescent cells against time. Data shown as mean ± SD (n = 3 biological replicates).


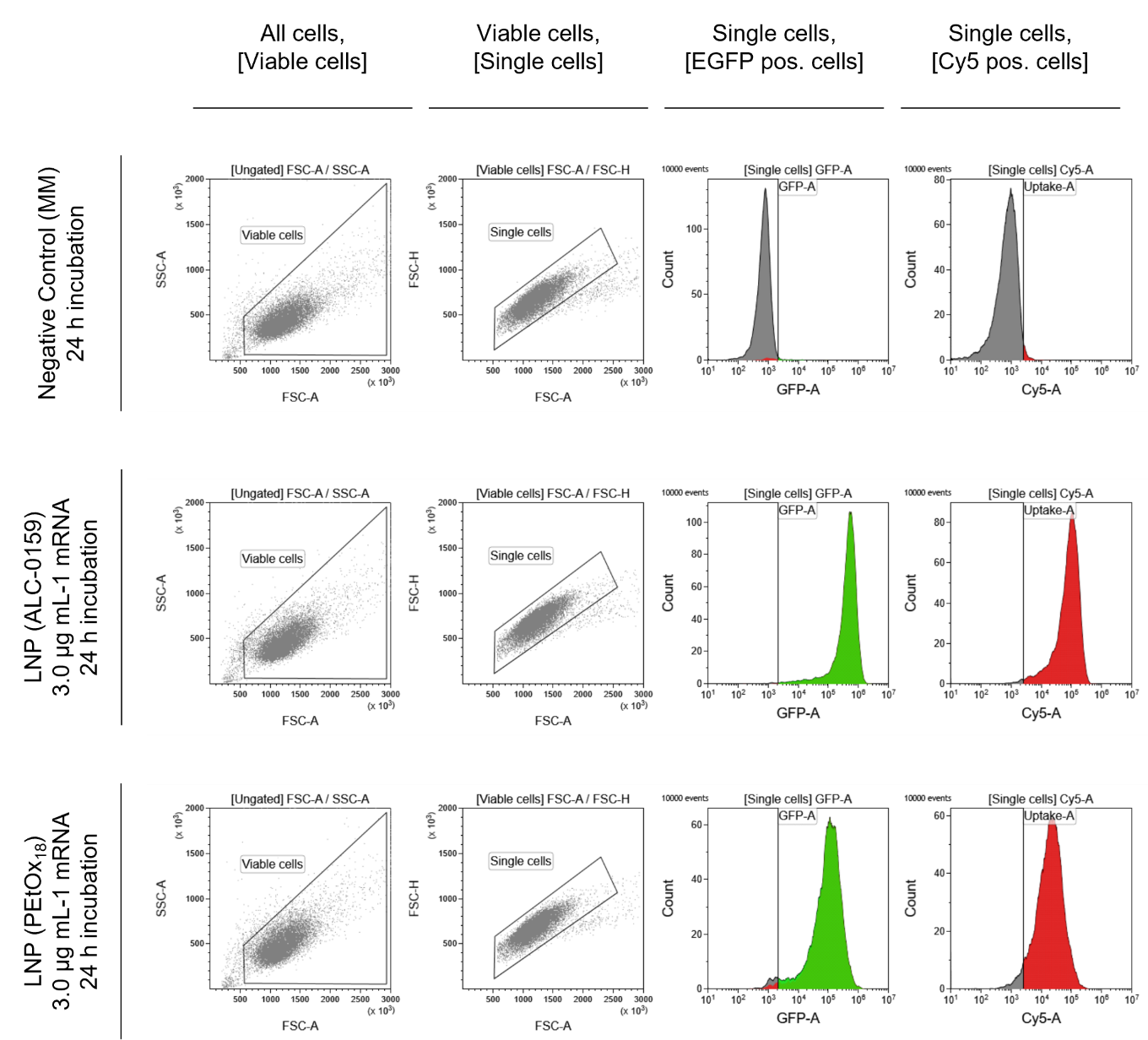


**Figure S 15:** Gating strategy: Uptake and transfection of HEK293T cells with mRNA (Cy5-EGFP). Viable single cells were analyzed by forward and sideward scatter (FSC/SSC). Fluorescence was measured at λ_Ex_ = 488 nm with a 525/40 nm bandpass filter (FITC channel) to detect EGFP and λ_Ex_ = 638 nm using a 660/10 nm bandpass filter to detect Cy5. Positive cells were identified by gating against the cells treated with only nucleic acid (MM). Analysis was conducted with Kaluza V 2.2.1.


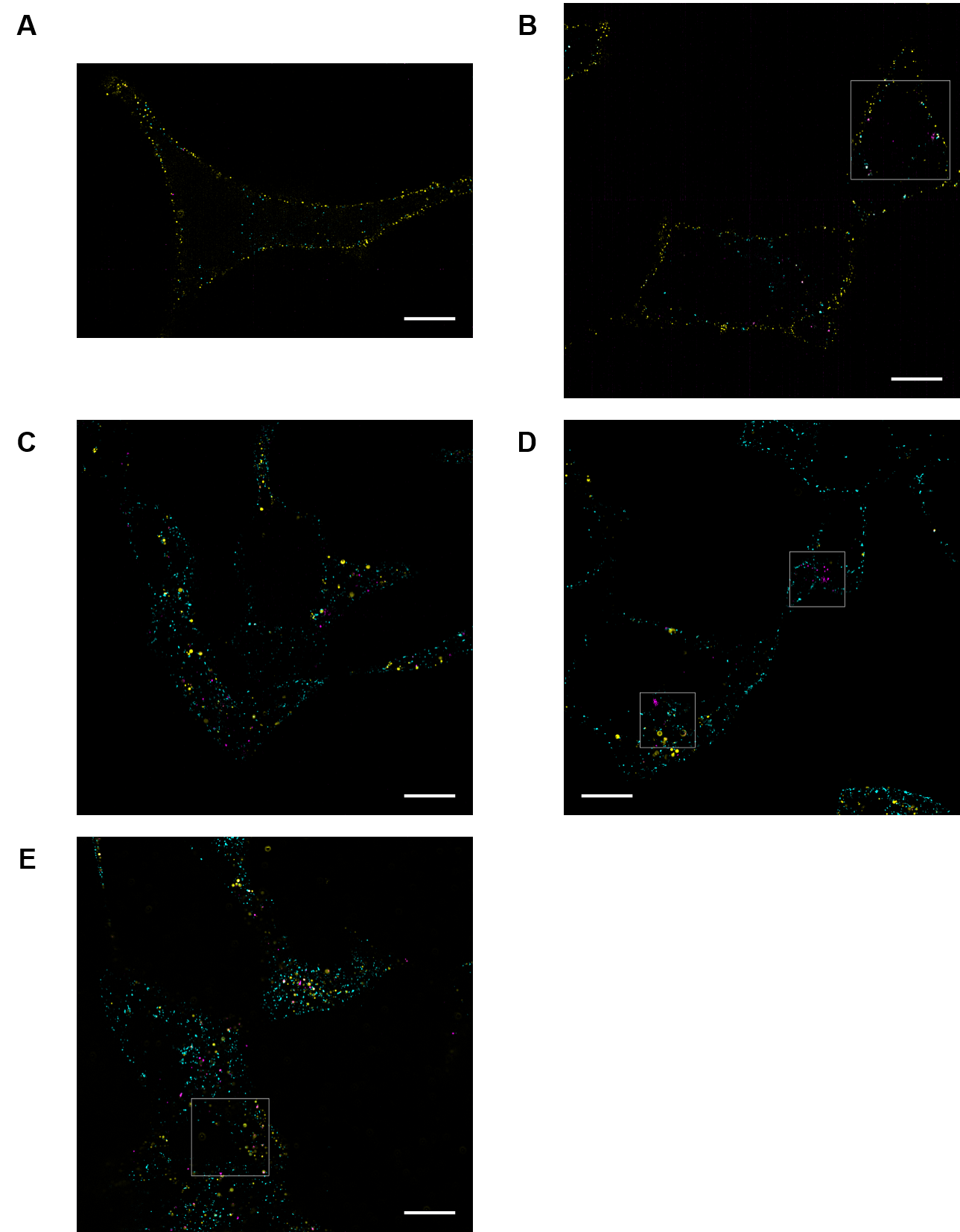


**Figure S 16:** Super-Resolution microscopy of HEK293T cells with mRNA loaded PEG LNPs (magenta) in context with endosomal cargo EGF (yellow) and Transferrin (cyan) at **A**: 1 min, **B**: 3 min, **C**: 30 min, **D**: 60 min and **E**: 120 min after incubation. Scale bars: 10 µm.


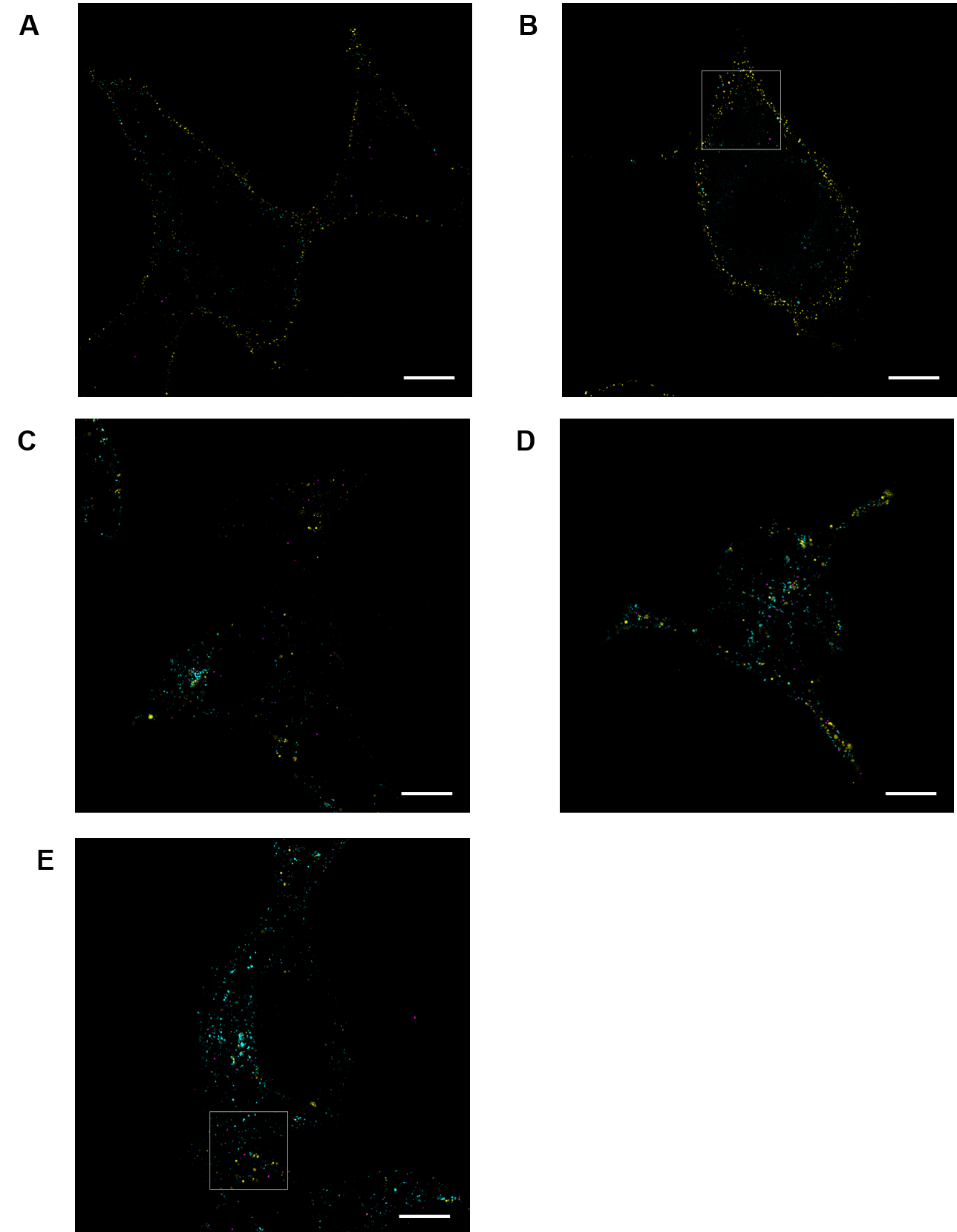


**Figure S 17:** Super-resolution microscopy of HEK293T cells with mRNA loaded PEtOx_18_ LNPs (magenta) in context with endosomal cargo EGF (yellow) and transferrin (cyan) at **A**: 1 min, **B**: 3 min, **C**: 30 min, **D**: 60 min and **E**: 120 min after incubation. Scale bars: 10 µm.
